# Developing a shared sepsis data infrastructure: a systematic review and concept map to FHIR

**DOI:** 10.1038/s41746-022-00580-2

**Published:** 2022-04-04

**Authors:** Emily B. Brant, Jason N. Kennedy, Andrew J. King, Lawrence D. Gerstley, Pranita Mishra, David Schlessinger, James Shalaby, Gabriel J. Escobar, Derek C. Angus, Christopher W. Seymour, Vincent X. Liu

**Affiliations:** 1 Department of Critical Care Medicine, University of Pittsburgh School of Medicine, Pittsburgh, PA USA; 2 Clinical Research, Investigation and Systems Modeling of Acute Illness (CRISMA) Center, Pittsburgh, PA USA; 3 Department of Emergency Medicine, University of Pittsburgh School of Medicine, Pittsburgh, PA USA; 4 Kaiser Permanente Division of Research, Oakland, CA USA; 5 Elimu Informatics, Richmond, CA USA; 6 Present Address: Assistant Professor of Critical Care and Emergency Medicine, University of Pittsburgh School of Medicine,, 200 Lothrop Street, #607, Pittsburgh, PA 15261 USA

**Keywords:** Diagnosis, Infectious diseases, Medical research

## Abstract

The development of a shared data infrastructure across health systems could improve research, clinical care, and health policy across a spectrum of diseases, including sepsis. Awareness of the potential value of such infrastructure has been heightened by COVID-19, as the lack of a real-time, interoperable data network impaired disease identification, mitigation, and eradication. The *Sepsis on FHIR* collaboration establishes a dynamic, federated, and interoperable system of sepsis data from 55 hospitals using 2 distinct inpatient electronic health record systems. Here we report on phase 1, a systematic review to identify clinical variables required to define sepsis and its subtypes to produce a concept mapping of elements onto Fast Healthcare Interoperability Resources (FHIR). Relevant papers described consensus sepsis definitions, provided criteria for sepsis, severe sepsis, septic shock, or detailed sepsis subtypes. Studies not written in English, published prior to 1970, or “grey” literature were prospectively excluded. We analyzed 55 manuscripts yielding 151 unique clinical variables. We then mapped variables to their corresponding US Core FHIR resources and specific code values. This work establishes the framework to develop a flexible infrastructure for sharing sepsis data, highlighting how FHIR could enable the extension of this approach to other important conditions relevant to public health.

## Introduction

Sepsis is among the deadliest medical conditions recognized worldwide. It occurs when infection triggers a dysregulated systemic immune response and can rapidly lead to organ failure and death^1^. Recent Global Burden of Disease estimates suggests that there were nearly 50 million cases in 2017 that resulted in 11 million deaths^2^. In the US, sepsis is the single most costly cause of hospitalization and contributes to as many as half of all hospital deaths^2,3^. Early diagnosis and treatment of infected patients with sepsis or high risk of progression to sepsis is imperative. Yet, sepsis diagnosis remains a major challenge due to several factors, including the absence of a gold standard test^4^, variability of patient presentation^5^, heterogeneity of illness progression^6^, among others^7–10^.

There is an urgent need to improve diagnostic excellence in sepsis^11^. One potential solution is the development of a flexible, scalable, and interoperable data infrastructure to screen and identify sepsis patients across health systems using real-time, granular clinical data available within electronic health records (EHRs)^11^. Despite clinical data for millions of sepsis or pre-sepsis patients routinely collected in EHRs, the healthcare enterprise has accessed only a small fraction of these data to improve the clinical understanding of sepsis. The development of a sepsis data backbone could be extended to meet diverse needs, including sepsis translational research, clinical care delivery, machine learning/artificial intelligence deployment, disease surveillance, quality improvement, and health policy. Furthermore, once established in sepsis, a similar data infrastructure could be tailored to include other conditions. For example, awareness of the potential value of this data infrastructure has been magnified by the COVID-19 pandemic—many COVID-19 inpatients met sepsis criteria with systemic inflammation, organ failure, and a high risk of mortality—highlighting the critical need for real-time data interoperability that could inform pandemic response, health system preparedness, translational research and clinical care^12–14^.

The Fast Healthcare Interoperability Resource (FHIR) Health Level Seven International (HL7) standard has shown great potential for modernizing the interchange of data through standardized FHIR resources and application programming interfaces (APIs) to access and exchange these data^15,16^. Indeed, FHIR has been endorsed by the Center for Medicare and Medicaid Services (CMS) and the Office of the National Coordinator for Health Information (ONC) as the preferred standard for EHR interoperability^17^.

In this study, we report on the development of *Sepsis on FHIR*, a framework to accelerate the interoperability of sepsis data across healthcare systems and EHRs. The work is a collaboration between Kaiser Permanente Northern California and the University of Pittsburgh Medical Center (UPMC) health systems to establish a dynamic, federated, and interoperable system of sepsis data from 55 diverse hospitals using two distinct inpatient EHR software systems. Here, we discuss phase 1 of *Sepsis on FHIR*. We first conducted a systematic review of the sepsis literature and identified a comprehensive set of relevant EHR clinical variables as a sharable set of features across sites. We then produced a concept mapping of clinical variables to FHIR resources. The resulting infrastructure can be adapted for the eventual expansion to other hospitals with different patient populations.

## Results

### Study characteristics

**Fig. 1:**
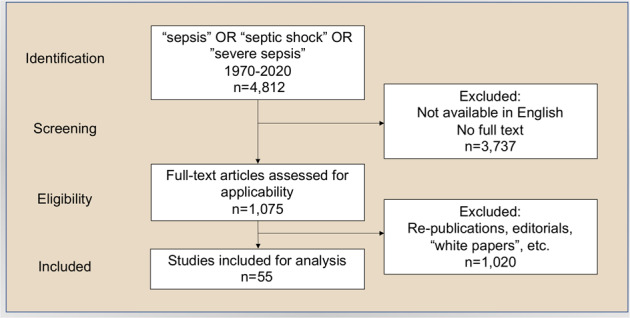
Flowchart for study selection. Initial searches identified 4812 manuscripts. After removing duplicate, non-English, non-full-text citations, 1075 full-text manuscripts were reviewed against inclusion criteria. After exclusion, 55 unique studies were included in the analysis.

We conducted a systematic review that retrieved 4812 citations; removing duplicate, non-English, non-full-text citations yielded 1075 full-text manuscripts (Fig. 1). After manual review, we excluded 1022 manuscripts that did not meet inclusion criteria or reported previously cited sepsis definitions, leaving 55 unique articles for analysis (Supplementary Data set 1). These included 8 consensus definitions, 7 systematic reviews and meta-analyses, 9 prospective cohort studies, 26 retrospective cohort studies, and 5 narrative literature reviews (Supplementary Fig. 1). All studies were reported between 1989 and 2020 (Supplementary Fig. 2).

The systematic review confirmed that several definitions and criteria were used to identify sepsis patients. Studies published prior to international consensus conference definitions identified sepsis patients using standardized criteria for patient enrollment used in trials performed by Bone et al.^18–21^. Organ dysfunction was defined using clinical markers of hypoperfusion including altered mentation, hypoxemia, elevated lactic acid, and oliguria^18^. Following the First International Consensus on Sepsis and Septic Shock, 16 studies analyzed defined sepsis patients using Systemic Inflammatory Response Syndrome (SIRS) criteria^6,20,22–35^. After the Sepsis-3 task force in 2016, 14 studies analyzed defined sepsis patients using the Sequential Organ Failure Assessment (SOFA) score^6,23–25,27,30–32,34–39^. Two studies defined organ dysfunction in sepsis patients using the logistic organ dysfunction score (LODS); two studies reported the multiple organ dysfunction syndrome (MODS)^21,22,40,41^. The Acute Physiologic Assessment and Chronic Health Evaluation (APACHE) score defined sepsis-associated organ dysfunction in eight analyzed studies^22,26,29,34,37,42–44^. Seven studies defined sepsis using claims-based definitions or International Classification of Diseases (ICD) standards^28,33,34,44–48^.

Definitions used to establish suspected or proven infection varied considerably. Nearly half (46%) of studies incorporated blood culture data, including body fluid sampling for culture or culture positivity. Many, however, relied on clinical suspicion as defined by the treating physician^18,34,35,41,43,49–55^. Two studies used clinical risk scores, including the clinical pulmonary infection score and infection probability score^35,43,56^.

### Data elements

We identified 788 clinical variables from the 55 manuscripts (Supplementary Table 3). Recorded data elements included clinical measurements (e.g., heart rate, respiratory rate, white blood cell count), infectious signs and symptoms (e.g., dysuria, abdominal pain), and individual ICD codes for diagnosis and procedures, current procedural terminology (CPT) and diagnosis-related groups (DRG) codes for sepsis, septicemia, and sepsis syndromes, organ dysfunction (e.g., acute kidney injury, hypotension) and infection (e.g., sepsis due to anaerobes, candidal sepsis) (Supplementary Table 3).

**Fig. 2:**
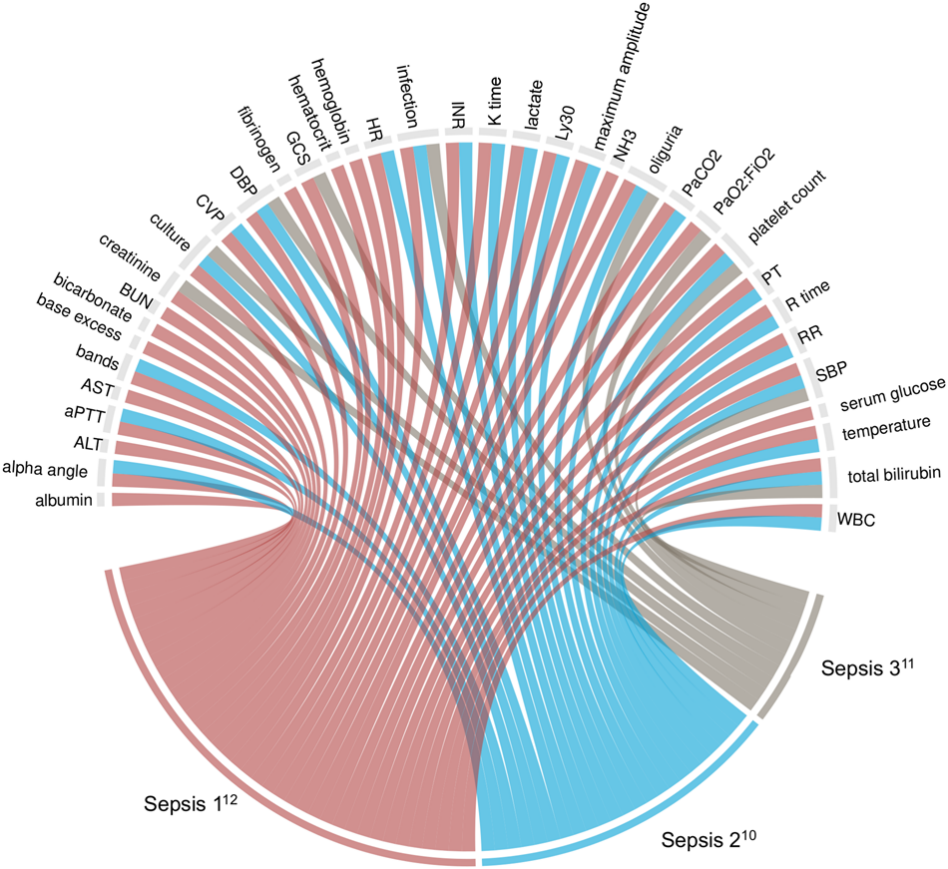
Chord diagrams showing the representation of clinical elements across international consensus definitions for sepsis. Ribbons connect from each of three international consensus definitions for sepsis to data elements. Only those data elements encompassed by sepsis consensus definitions are displayed. *Abbreviations:* ALT alanine aminotransferase, aPTT activated partial thromboplastin time, AST aspartate aminotransferase, BUN blood urea nitrogen, CVP central venous pressure, DBP diastolic blood pressure, GCS Glasgow coma scale score, HR heart rate, INR international normalized ratio, Ly30 lysis in 30 minutes, NH3 ammonia level, PaCO_2_ partial pressure of arterial carbon dioxide, FiO_2_ fraction of inspired oxygen, PT prothrombin time, RR respiratory rate, SBP systolic blood pressure, WBC white blood cell count.

After the removal of duplicates and unstructured variables, 151 unique clinical variables remained (Supplementary Table 4). Variables represented 7 broad domains including, patient characteristics, vital signs and laboratory tests, interventions data (e.g., medication administration, diagnostic tests, catheterization, surgical events), fluid balance, location information, healthcare use and outcomes, and administrative/billing codes. All major sepsis consensus definitions were represented by selected clinical variables, capturing the evolution of sepsis definitions over time (Fig. 2).

**Fig. 3:**
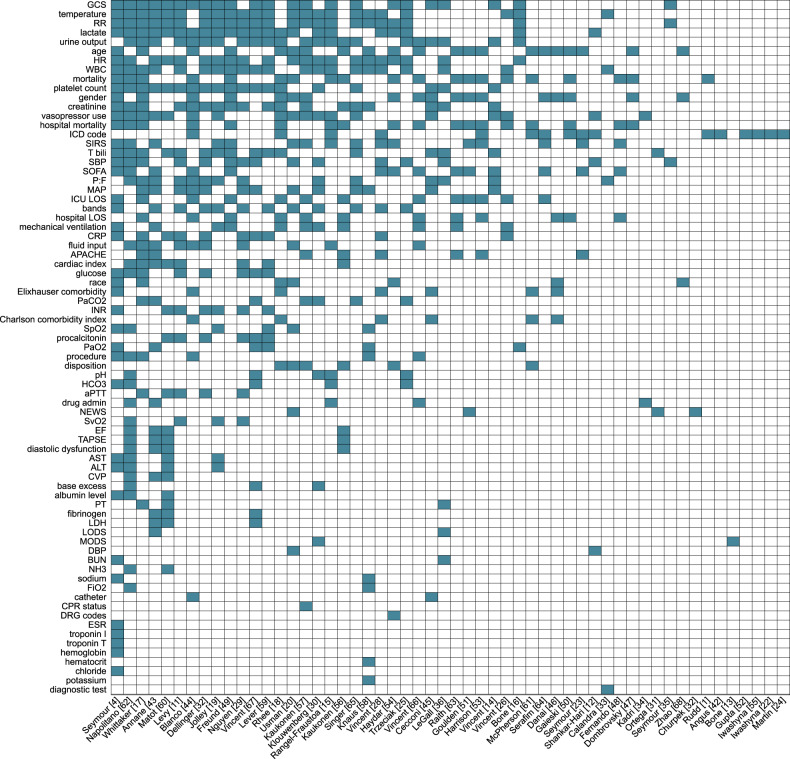
Data elements reported by a manuscript in heatmap. Data elements are displayed on the *y* axis; manuscripts included in analysis displayed on *x* axis. Axes are sorted so variables most frequently cited are grouped together on the left, whereas manuscripts contributing infrequently reported variables are grouped on the right. Blue shading represents the data element reported by the manuscript, whereas data elements not reported by the manuscript are not shaded. RR respiratory rate, GCS Glasgow Coma Scale score, WBC white blood cell count, HR heart rate, T Bili, total bilirubin, SIRS systemic inflammatory response syndrome, SBP systolic blood pressure, P:F ratio of partial pressure of arterial oxygen to fraction of inspired oxygen, ICD international classification of diseases, SOFA sequential organ failure assessment score, ICU intensive care unit, LOS length of stay, MAP mean arterial pressure, CRP c-reactive protein, APACHE acute physiology and chronic health enquiry, INR international normalized ratio, PaCO_2_ partial pressure of arterial carbon dioxide, SpO_2_ oxygen saturation, aPTT activated partial thromboplastin time, HCO_3_ bicarbonate, ALT alanine aminotransferase, AST aspartate aminotransferase, TAPSE tricuspid annular plane systolic excursion, EF ejection fraction, SvO_2_ saturation of venous oxygen, NEWS national early warning system, LDH lactate dehydrogenase, PT prothrombin time, CVP central venous pressure, FiO_2_ fraction of inspired oxygen, NH_3_ ammonia level, BUN blood urea nitrogen, DBP diastolic blood pressure, MODS multiple organ dysfunction syndrome, LODS logistic organ dysfunction syndrome, ESR erythrocyte sedimentation rate, DRG diagnosis-related groups.

The most frequently reported patient demographic characteristics included age, sex, and measures of comorbidity (Fig. 3). Vital signs and laboratory tests were both used for sepsis definitions and to describe baseline characteristics. Infection was most often described by body fluid sampling for culture and white blood cell count. Organ dysfunction was most commonly described using respiratory rate, temperature, Glasgow Coma Scale (GCS) score, and urine output. Other variables, including troponin, albumin level, and erythrocyte sedimentation rate, were reported only once, but were considered critical for defining specific sepsis subtypes^6^.

### FHIR mapping

We mapped the 151 clinical variables to their corresponding FHIR resources and, where appropriate, to specific code values that represented each element (Supplementary Table 5). Variables mapped most frequently to the FHIR DiagnosticReport resource (41%), followed by the observation (33%), patient (12%), encounter (9%), or procedure (5%) resources (Supplementary Table 5).

To promote flexibility, we linked variables to several FHIR resource types whenever possible. For example, the variable “serum sodium” is represented as a FHIR Observation resource where the Observation.code data element is mapped to the Logical Observation Identifiers Names and Codes (LOINC) code 2951-2 (referring to “Sodium [Moles/volume] in Serum or Plasma”). LOINC is the most commonly used international healthcare terminology standard which describes a reference set of health data and codes for laboratory and clinical observations. FHIR mappings frequently link to more than one LOINC code. For example, blood urea nitrogen (BUN) mapped to both LOINC codes 3094-0 (BUN SerPl-mCnc) and 6299-2 (BUN Bld-mCnc). For variables linked to more than one code, we established a HL7 FHIR-based sepsis value set to represent a set of codes with the same clinical meaning. Most variables (99 out of 151) were FHIR resource observations with direct mappings to LOINC codes. In contrast, the variable “Admit Time” mapped directly to an existing FHIR data element for healthcare encounter time stamps (Encounter.period.start).

We also identified some variables that require additional computation. For example, “Days of Vasopressors”—the duration of treatment with vasoactive agents to support blood pressure—requires calculation using variables mapped to other resources. The start and end dates of vasopressor therapy, which are represented as the FHIR MedicationAdministration resource, are required. In addition, these data may also be represented by RxNORM codes, the US reference terminology standard representing vasopressors (e.g., epinephrine, norepinephrine).

Taken together, clinical variables were successfully mapped to US Core FHIR elements, LOINC, and RxNorm codes to ensure and enhance generalizability and flexibility across health systems and EHR.

## Discussion

In phase 1 of *Sepsis on FHIR*, we conducted a systematic review of 55 studies, identifying 151 variables relevant to sepsis diagnosis, sepsis definitions and subtyping. We then mapped these variables to a data standard (FHIR) endorsed by national healthcare entities^17^. The goal of this work is not to define a final or complete set of sepsis diagnostic and clinical criteria, nor to develop a data interoperability resource limited only to two large US healthcare systems. Instead, we aim to establish a publicly available *Sepsis on FHIR* resource that will promote the use of a flexible, scalable, federated, and interoperable source of clinical data that can be contributed to and used by many stakeholders. Our work could be easily applied to other clinical conditions.

This work focused on sepsis as it is common, costly, and deadly, with significant opportunities to improve treatment and outcomes^2^. Sepsis treatment can be challenging due to a number of factors, however, the key step to effective treatment is early diagnosis. Timely diagnosis is the critical factor determining patient outcomes, since delays in diagnosis (with concomitant delayed treatment with antibiotics, resuscitation, and organ system stabilization) are associated with increased mortality^7,57^. Numerous leading entities, including the National Institute of General Medical Sciences and the National Academy of Medicine, have identified the lack of standardized and interoperable data resources as a critical limitation to improving sepsis diagnosis and treatment^6,58^.

Despite the availability of millions of EHR records detailing patients’ presentation, organ failure, pathogen, treatment, and outcomes today, the lack of access to these data in aggregate stymie further progress^59^. Multicenter data may be available in some instances, such as within networks of hospitals linked by a single EHR software vendor or within larger health systems or research consortia, for example, but there is a lack of a standardized resource for diverse stakeholders who wish to contribute to or access these data. Larger registries of sepsis data exist through state or national efforts, however, these comprise a relatively narrowly-defined set of criteria targeted to performance or quality improvement efforts^60^. Further, the cost of maintaining these data has also raised alarm among entities subject to reporting requirements^61,62^.

The development of an automated, flexible infrastructure for sharing sepsis data represents a key step toward achieving diagnostic excellence and improving sepsis outcomes for several reasons. First, compiling high-fidelity health data from millions of patient encounters creates a substantial opportunity to uncover sepsis subgroups^63^. Recent studies have highlighted the heterogeneity in treatment effects among specific subgroups which demonstrates potentially adverse effects of current treatment approaches in some patient subtypes^6,64^. Thus, establishing representative subtypes and identifying them in real-time will be key for initiating clinical trials and targeting treatments. Second, while focused, small repositories of biospecimens exist in sepsis, they cannot be easily appended to detailed clinical data shared across sites to improve their utility and statistical power^65^. Third, as described above, current reporting requirements for the CMS SEP-1 measure is highly resource-intensive and of uncertain benefit for patients^62^. Automated extraction of patient data can improve reporting while also helping to refine the use of policy-driven targets. Fourth, while there are many emerging machine learning and artificial intelligence algorithms designed to improve the prediction of sepsis onset or deterioration, they can be brittle (i.e., predictive performance degrades) when developed in one healthcare context and exported to an external context^66^. A federated learning system built using data from many diverse environments can improve the generalizability, representativeness, and transparency of such algorithms for clinical care. Fifth, as evident in the COVID-19 pandemic, the lack of a national real-time system of interoperable data substantially hampers the identification, mitigation, and eradication of large-scale communicable diseases^12^. Finally, the extension of this platform beyond the United States may allow for standardized international data exchange.

There are many steps that logically follow from this work. Using the Sepsis on FHIR foundation, our two health systems will instantiate FHIR repositories at each site while continuing work to overcome several barriers. Among these continuing barriers, the most important are the instability of the EHRs (in which codes are frequently changed/altered), persistence of data elements that are unmapped, and difficulties in bulk extraction of FHIR-mapped data elements. For example, real-time extraction of FHIR data in sepsis can be costly and, thus, shadow or parallel data systems that offload computation to non-operational systems may be needed to ensure the availability of data relevant to clinical care or trials. In addition, data elements collected serially will need to be refreshed at a clinically relevant tempo (Supplementary Table 5).

Further, several key issues involving data governance, privacy, and security must be addressed prior to the aggregation of any patient data. To address data governance and privacy, the development of a federated data infrastructure (i.e., local data that never leave an individual system) reduces the risk associated with data transmission. The FHIR standard supports key interoperability enablers that can support a federated analytics platform^67^. To ensure data security, FHIR also supports secure exchange of clinical data and the ability to populate powerful standards-based research analytics platforms such as PCORNET Common Data Model (CDM)^68^. Deidentified FHIR-based data can be used to populate CDMs at each local site that can in turn support federated queries across multiple sites against the deidentified data in the CDM instances.

Scalability beyond the originating health systems can also be challenging given the need for additional resources for initiation and maintenance. Thus, to enhance standardization for data exchange, FHIR Sepsis Implementation Guides (IG) can be developed and balloted for general use^69^. FHIR IGs constrain the standard to meet specific use cases for interoperability and are the cornerstone of FHIR interoperability. IGs contain profiles for each resource that specify which terminology value sets should be used for valid messaging of observational data between systems (e.g., which LOINC values are used to map clinical variables). Value sets leverage a change management and governance process that is a well-established part of the Value Set Authority Center (VSAC), a nationally available National Library of Medicine repository. This includes the submission, review and publishing process used to manage requests to expand or change value set content over time. The endorsed value sets can also be maintained and distributed widely through the VSAC as a national portal. Once established, FHIR profiles can easily reference VSAC value sets through a process known as terminology binding. Clinical decision support and analytic tools can be standardized through these nationally cataloged VSACs.

In addition to these challenges, we recognize several limitations in this study. First, we only identified structured clinical variables used in prior studies and already available in the EHR to define sepsis and sepsis subgroups. Other data that are likely to be valuable to sepsis diagnosis and subgrouping such as clinician unstructured documentation, radiographic results, biomarkers or other -omic data, or pathogenic variables were not identified and included. The value of a flexible *Sepsis on FHIR* approach, however, is that such data can be added incrementally to the existing resources as they become available or central to sepsis management. Second, we recognize several limitations in using FHIR for data interoperability: (1) clinical variables were mapped to best-fit FHIR resources, (2) not all clinical data variables were mapped to a FHIR resource, and (3) not all EHR vendors are implementing the same FHIR APIs, potentially limiting expansion to other health systems. Finally, though literature summarized in the systematic review is from across the globe and selected variables mapped to FHIR standards are widely applicable, we focus on a U.S. EHR landscape. Future work to expand Sepsis on FHIR to international sites is needed.

In conclusion, we identified 151 clinical variables from 55 manuscripts needed to define sepsis and sepsis subgroups. We mapped these variables to established FHIR resource standards. This work represents the first step towards a real-time, federated, and flexible *Sepsis on FHIR* clinical data resource which can form the foundation needed for interoperability of data across healthcare systems to improve sepsis diagnostic excellence and outcomes.

## Methods

The Institutional Review Boards of Kaiser Permanente Northern California (1533936) and the University of Pittsburgh approved the study (STUDY 20020141).

### Study overview

The study objective was to conduct a systematic literature review to identify a core set of variables needed to define sepsis and its subtypes, then, map these variables onto existing FHIR standards. We sought to first identify and map a core set of variables that have been historically used to define sepsis and its subtypes. However, we anticipate expansion of this flexible, foundational framework in response to the future evolution of sepsis care.

We conducted the literature search in three steps. First, we identified studies describing a consensus sepsis definition. Second, we identified studies in which a sepsis, severe sepsis, or septic shock cohort was defined, cataloging variables needed to identify these patient groups based on the multiple sepsis definitions in common use today. Finally, we identified clinical variables needed to perform detailed subtyping of sepsis patients based on currently published studies describing sepsis subtypes. When reviewing the studies, we also selected variables known to be associated with adverse outcomes for sepsis, including the source of infection (e.g., pneumonia vs urinary tract infection), the severity of organ dysfunction (e.g., presence of hypotension or altered mental status), need for organ support treatments (e.g., mechanical ventilation or dialysis) and other treatment modalities (e.g., corticosteroids). Once variables were identified, we assigned each to an existing FHIR resource, the common building blocks used to define and exchange specific EHR data. This review followed guidance published by the Cochrane collaboration and conforms to the Preferred Reporting Items for Systematic Reviews and Meta-Analyses (PRISMA) standards^70,71^. The study protocol was not prospectively registered.

This phase represents the key first steps in the development of *Sepsis on FHIR*, a framework to accelerate the interoperability of a dynamic, and federated system of sepsis data across healthcare systems and EHRs (Supplementary Fig. 3).

### Systematic review

We sought to identify relevant studies that (1) describe consensus sepsis definitions, (2) provide criteria for sepsis, severe sepsis, septic shock or a sepsis subgroup, or (3) detail sepsis subtyping. Search methods were designed following guidance published by the Cochrane collaboration^70^. This systematic review conforms all these criteria.

To develop the list of search terms, we collated applicable MeSH terms provided for each major Sepsis consensus definition (i.e., Sepsis-1^40^, Sepsis-2^72^, and Sepsis-3^32^). These included “sepsis”, “septic shock”, “severe sepsis”. Natural language search terms included “definition”, “decision rule”, “diagnosis”, “characteristics”, “variables”, “criteria”, and “epidemiology”. Boolean operators OR and AND for combining search term(s) were used to streamline the procedure. Next, we employed the PICOS (population, intervention, comparator, outcome, and study design) search strategy, a widely cited tool for synthesizing scientific research^73^. By using the PICOS structure, we sought to identify studies reporting on sepsis patient Populations to elucidate sepsis cohort definitions used.

We sought original research published in peer-reviewed journals reporting on adult patients with sepsis, severe sepsis, and/or septic shock. Selected study designs included consensus statements, systematic reviews, meta-analyses, randomized clinical trials, case-control studies, and cohort studies. Studies not written in English, published prior to 1970, reporting pediatric populations or animals, or “gray” literature were prospectively excluded.

We searched English language studies published from January 1, 1970 through January 31, 2020. We systematically searched three databases: PubMed, MEDLINE, and the Cochrane Library. Search strategies were developed for each database, starting with PubMed then adapted for each subsequent search (Supplementary Tables 1 and 2). We screened all titles and abstracts returned from searches against inclusion criteria and removed duplicate studies. Reference lists from selected manuscripts were scrutinized by two authors (E.B. and J.K.) to identify additional studies meeting selection criteria. Manuscripts were reviewed and ranked by two independent reviewers (E.B. and J.K). Full-text versions were then obtained and independently reviewed by co-authors. Discrepancies throughout the review process were resolved through discussion with study senior authors (C.S. and V.L.) Initial searches were conducted June 2019 with a second search completed at the end of January 2020.

### Variable selection

We collected specific study characteristics (e.g., title, authors, year of publication, journal, study design); population characteristics (e.g., demographics, admission diagnosis, level of care); clinical variables (e.g., vital signs, laboratory measurements); organ support (e.g., provision of mechanical ventilation or dialysis); treatments (e.g., corticosteroids), and outcomes (e.g., 28-day mortality, intensive care unit (ICU) admission, hospital and/or ICU length of stay) using a standardized data extraction form developed in Microsoft Word (Supplementary Table 3). After removing duplicates, the final list of variables was independently reviewed by members of the study team (E.B., J.K., V.L., and C.S.). We also convened a stakeholder group with expertise in sepsis clinical care, quality improvement, patient and family experience, EHR-based research, and healthcare interoperability to adjudicate the final list of clinical variables for completeness and applicability (Supplementary Note 1).

### FHIR mapping

After compiling the list of variables, we then assigned each a FHIR resource data element to each (Supplementary Fig. 4). A resource is the building block in the FHIR standard, providing a common way to define and represent all exchangeable data^74^. A FHIR resource is composed of data elements that define the content of that resource. For example, a clinical observation resource is defined by data elements such as the date of the observation, the type of observation (e.g., a vital sign, laboratory measurement), the value and units of measure. US FHIR resources are constrained to which data elements are required to exchange patient data in the US; these constraints are called US Core FHIR profiles. Example US Core FHIR profiles include vital signs, laboratory measurements, conditions or problems, medications, and other key data element types.

To share clinical data across health systems, variables from the EHR must be mapped onto existing FHIR resources to the data element level. This was done in two steps. First, each clinical variable was reviewed against FHIR documentation for a corresponding resource data element^75–89^. Next, each variable was fit within an existing FHIR resource deemed best-fit for the source concept.

To ensure generalizability across health systems and EHR, we first attempted to find US Core FHIR Profile mappings and if they were unavailable, we mapped data elements to the FHIR base resources.

### Reporting summary

Further information on research design is available in the Nature Research Reporting Summary linked to this article.

## Supplementary information

Supplemental File

Dataset 1

Reporting Summary Checklist

## Data Availability

Aggregate data supporting the findings of this article are cataloged in Supplementary Table 5.
