# Supplementary material for: Developing a shared sepsis data infrastructure: a systematic review and concept map to FHIR
Source: NPJ Digit Med. 2022 Apr 4;5:44. doi: 10.1038/s41746-022-00580-2 (PMC8979949; doi:10.1038/s41746-022-00580-2)
Supplement: Supplementary file 1 — Supplemental File [file 41746_2022_580_MOESM1_ESM.pdf]

Supplementary Content

Developing a shared sepsis data infrastructure: a systematic review and concept map to FHIR

	**Page**
**Supplementary Note** 1. List of collaborators	2
**Supplementary Figure** 1. Bar graph of number of manuscripts analyzed per study type	3
**Supplementary Figure** 2. Histogram of studies by publication year	4
**Supplementary Figure** 3. Sepsis on FHIR project schematic	5
**Supplementary Figure** 4. FHIR mapping schematic	6
**Supplementary Table** 1**.** PubMed search query	7
**Supplementary Table** 2. MEDLINE search query	9
**Supplementary Table 3.** Master list of cataloged clinical variables	10
**Supplementary Table 4**. Final list of clinical variables	30
**Supplementary Table 5**. Integrated clinical variables and FHIR resources	32
**Supplementary References**	45

Supplementary Note 1**:** List of Collaborators

We would like to acknowledge the contributions of the following advisory board members:

- Timothy Buchman, MD PhD; Professor of Surgery and Anesthesiology, Emory University; Senior Advisor for the Biomedical Advanced Research and Development Authority (BARDA)
- Ciaran and Orlaith Staunton of The Rory Staunton Foundation (a prominent patient advocacy organization for sepsis awareness)
- Julia Adler-Milstein, PhD; Associate Professor, UCSF (an expert in health care interoperability)
- Marcus Freirich, MD MBA; Chief Medical Officer of the NY State Department of Health
- Sanjay Doddamani, MD MBA; Medical Officer, Center for Medicare and Medicaid Innovation
- Tim Uyeki, MD MPH; Chief Medical Officer for the Influenza Division, Center for Disease Control
- Laura Evans, MD; Surviving Sepsis Guidelines Co-Chair; Medical Director Critical Care, NYU

Supplementary Figure 1. Bar graph of number of manuscripts analyzed per study type (N=55)

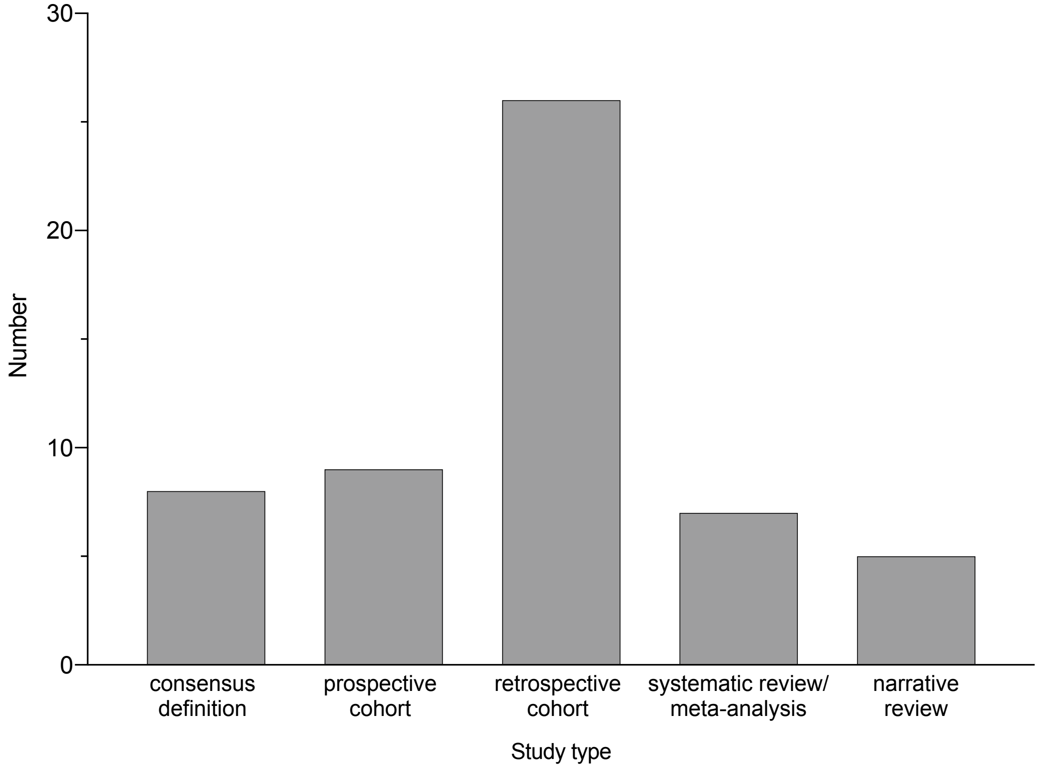

Supplementary Figure 2. Histogram of studies by publication year (N=55)

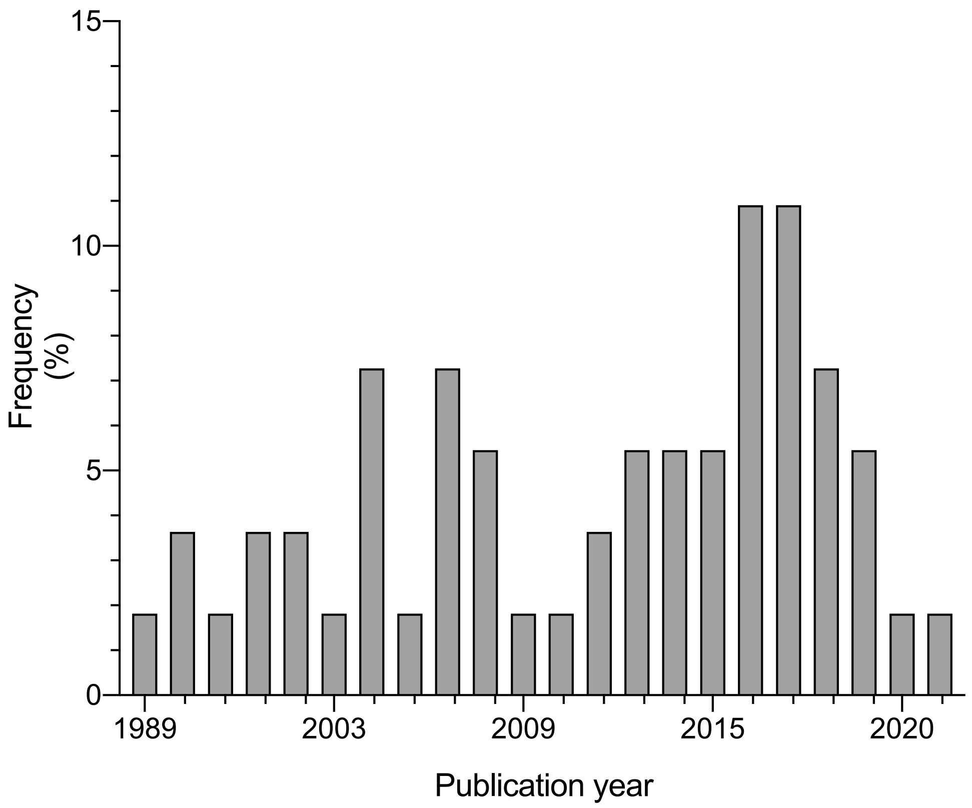

Supplementary Figure 3. Sepsis on FHIR project schematic

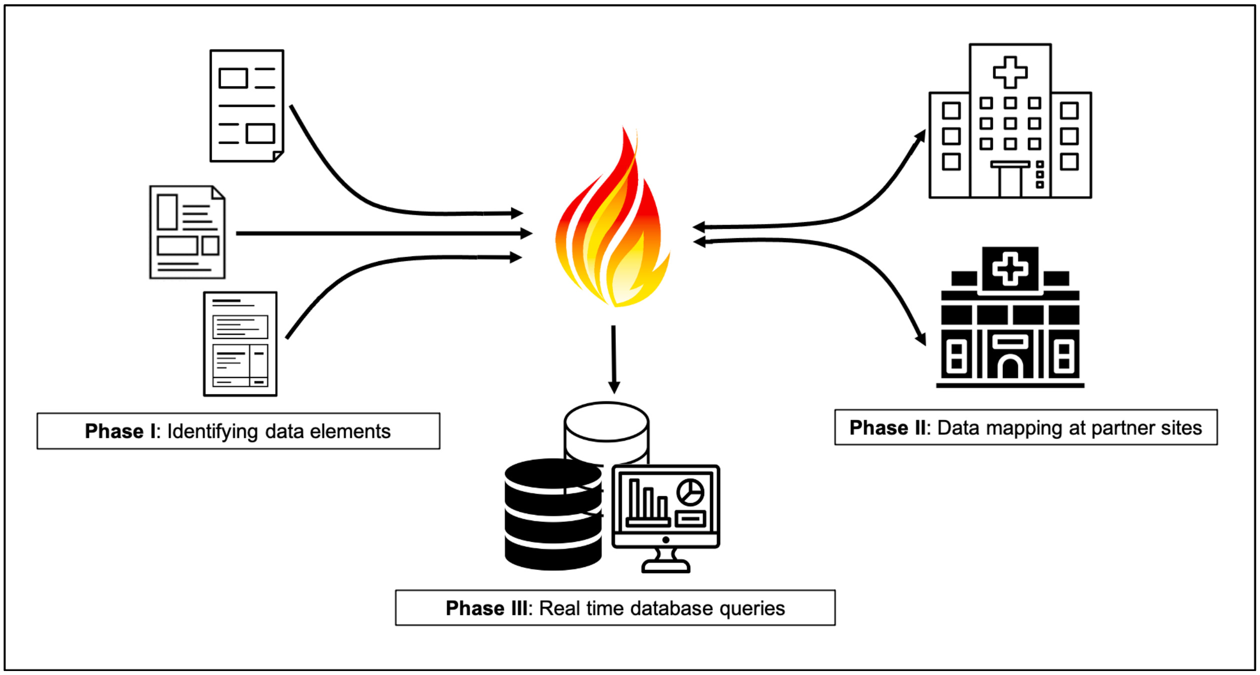

Schematic illustrates the 3 phases of the *Sepsis on FHIR* project. Phase I includes a systematic review to identify clinical variables required to define sepsis and its subtypes and produce a concept mapping of elements onto Fast Healthcare Interoperability Resources (FHIR). In phase II, UPMC and KPNC will perform data mapping specific to individualized EHR system. Once variables from each site are mapped to FHIR resources, Phase III will involve development of real time database queries across health systems.

Supplementary Figure 4. FHIR mapping schematic

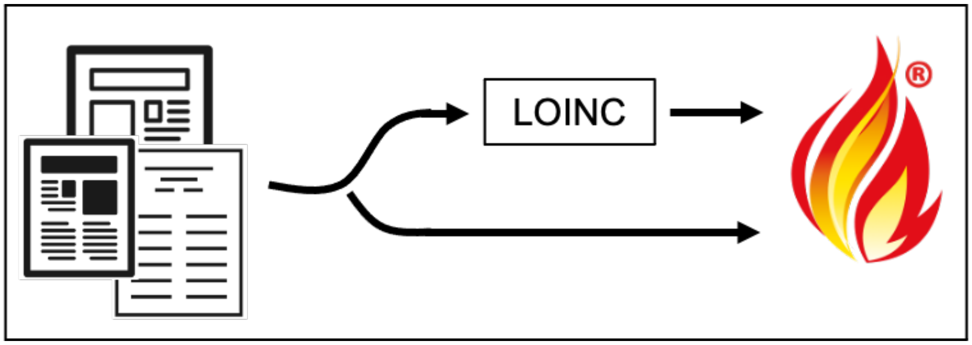

Schematic describes FHIR mapping steps. Clinical variables were mapped to either directly FHIR resources or Logical Observation Identifiers Names and Codes (LOINC).

Interpretative example: The variable ‘serum sodium’ is represented as a FHIR Observation resource where the Observation.code data element is mapped to the Logical Observation Identifiers Names and Codes (LOINC) code 2951-2 (referring to “Sodium [Moles/volume] in Serum or Plasma”).

Most variables (99 out of 151) were FHIR resource observations with direct mappings to LOINC codes. In contrast, the variable “Admit Time” mapped directly to an existing FHIR data element for healthcare encounter time stamps (Encounter.period.start) as opposed to a LOINC-based observation.

Supplementary Table 1. PubMed search query

1	("sepsis"[Title/Abstract] AND "septic shock"[Title/Abstract] AND "severe sepsis"[Title/Abstract]) AND (1974:2020[pdat]); (3,710)
2	("sepsis"[Title/Abstract] AND "septic shock"[Title/Abstract] AND "severe sepsis"[Title/Abstract] AND 1974/01/01:2020/12/31[Date - Publication]) AND (1974:2020[pdat]); (1,459)
3	("sepsis"[Title/Abstract] AND "septic shock"[Title/Abstract] AND "severe sepsis"[Title/Abstract] AND 1974/01/01:2020/12/31[Date - Publication] AND ("loattrfree full text"[Filter] AND 1974/01/01:2020/12/31[Date - Publication]) AND ("definite"[All Fields] OR "definitely"[All Fields] OR "definition"[All Fields] OR "definitional"[All Fields] OR "definitions"[All Fields] OR "definitive"[All Fields] OR "definitively"[All Fields])) AND (ffrft[Filter]); (117)
4	("sepsis"[Title/Abstract] AND "septic shock"[Title/Abstract] AND "severe sepsis"[Title/Abstract] AND 1974/01/01:2020/12/31[Date - Publication] AND ("loattrfree full text"[Filter] AND 1974/01/01:2020/12/31[Date - Publication]) AND (("decision"[All Fields] OR "decision s"[All Fields] OR "decisions"[All Fields] OR "decisive"[All Fields] OR "decisively"[All Fields]) AND "rule"[All Fields]) AND "loattrfree full text"[Filter]) AND (ffrft[Filter]); (4)
5	("sepsis"[Title/Abstract] AND "septic shock"[Title/Abstract] AND "severe sepsis"[Title/Abstract] AND 1974/01/01:2020/12/31[Date - Publication] AND ("loattrfree full text"[Filter] AND 1974/01/01:2020/12/31[Date - Publication]) AND ("diagnosable"[All Fields] OR "diagnosi"[All Fields] OR "diagnosis"[MeSH Terms] OR "diagnosis"[All Fields] OR "diagnose"[All Fields] OR "diagnosed"[All Fields] OR "diagnoses"[All Fields] OR "diagnosing"[All Fields] OR "diagnosis"[MeSH Subheading]) AND "loattrfree full text"[Filter] AND (ffrft[Filter]); (779)
6	("sepsis"[Title/Abstract] AND "septic shock"[Title/Abstract] AND "severe sepsis"[Title/Abstract] AND 1974/01/01:2020/12/31[Date - Publication] AND ("loattrfree full text"[Filter] AND 1974/01/01:2020/12/31[Date - Publication]) AND ("characteristic"[All Fields] OR "characteristics"[All Fields]) AND "loattrfree full text"[Filter] AND (ffrft[Filter]); (202)
7	("sepsis"[Title/Abstract] AND "septic shock"[Title/Abstract] AND "severe sepsis"[Title/Abstract] AND 1974/01/01:2020/12/31[Date - Publication] AND ("loattrfree full text"[Filter] AND 1974/01/01:2020/12/31[Date - Publication]) AND ("variabilities"[All Fields] OR "variability"[All Fields] OR "variable"[All Fields] OR "variable s"[All Fields] OR "variables"[All Fields] OR "variably"[All Fields]) AND "loattrfree full text"[Filter] AND (ffrft[Filter]); (183)

8	("sepsis"[Title/Abstract] AND "septic shock"[Title/Abstract] AND "severe sepsis"[Title/Abstract] AND 1974/01/01:2020/12/31[Date - Publication] AND ("loattrfree full text"[Filter] AND 1974/01/01:2020/12/31[Date - Publication]) AND ("criteria s"[All Fields] OR "criterias"[All Fields] OR "standards"[MeSH Subheading] OR "standards"[All Fields] OR "criteria"[All Fields]) AND "loattrfree full text"[Filter] AND (ffrft[Filter]); (280)
9	("sepsis"[Title/Abstract] AND "septic shock"[Title/Abstract] AND "severe sepsis"[Title/Abstract] AND 1974/01/01:2020/12/31[Date - Publication] AND ("loattrfree full text"[Filter] AND 1974/01/01:2020/12/31[Date - Publication]) AND ("epidemiologies"[All Fields] OR "epidemiology"[MeSH Subheading] OR "epidemiology"[All Fields] OR "epidemiology"[MeSH Terms] OR "epidemiology s"[All Fields]) AND "loattrfree full text"[Filter] AND (ffrft[Filter]); (585)

Supplementary Table 2. MEDLINE search query

1	("sepsis"[Title/Abstract] AND "severe sepsis"[Title/Abstract] AND "septic shock"[Title/Abstract]) AND (1974:2020[pdat]); (3,710)
2	("sepsis"[Title/Abstract] AND "severe sepsis"[Title/Abstract] AND "septic shock"[Title/Abstract]) AND ((medline[Filter]) AND (1974:2020[pdat])); (3,267)
3	("sepsis"[Title/Abstract] AND "severe sepsis"[Title/Abstract] AND "septic shock"[Title/Abstract] AND ("medline"[Filter] AND 1974/01/01:2020/12/31[Date - Publication]) AND ("definite"[All Fields] OR "definitely"[All Fields] OR "definition"[All Fields] OR "definitional"[All Fields] OR "definitions"[All Fields] OR "definitive"[All Fields] OR "definitively"[All Fields])) AND (medline[Filter]); (205)
4	("sepsis"[Title/Abstract] AND "severe sepsis"[Title/Abstract] AND "septic shock"[Title/Abstract] AND ("medline"[Filter] AND 1974/01/01:2020/12/31[Date - Publication]) AND (("decision"[All Fields] OR "decision s"[All Fields] OR "decisions"[All Fields] OR "decisive"[All Fields] OR "decisively"[All Fields]) AND "rule"[All Fields]) AND "medline"[Filter]); (6)
5	("sepsis"[Title/Abstract] AND "severe sepsis"[Title/Abstract] AND "septic shock"[Title/Abstract] AND ("medline"[Filter] AND 1974/01/01:2020/12/31[Date - Publication]) AND ("diagnosable"[All Fields] OR "diagnosi"[All Fields] OR "diagnosis"[MeSH Terms] OR "diagnosis"[All Fields] OR "diagnose"[All Fields] OR "diagnosed"[All Fields] OR "diagnoses"[All Fields] OR "diagnosing"[All Fields] OR "diagnosis"[MeSH Subheading]) AND "medline"[Filter]); (1,948)
6	("sepsis"[Title/Abstract] AND "severe sepsis"[Title/Abstract] AND "septic shock"[Title/Abstract] AND ("medline"[Filter] AND 1974/01/01:2020/12/31[Date - Publication]) AND ("characteristic"[All Fields] OR "characteristics"[All Fields]) AND "medline"[Filter]); (391)
7	("sepsis"[Title/Abstract] AND "severe sepsis"[Title/Abstract] AND "septic shock"[Title/Abstract] AND ("medline"[Filter] AND 1974/01/01:2020/12/31[Date - Publication]) AND ("variabilities"[All Fields] OR "variability"[All Fields] OR "variable"[All Fields] OR "variable s"[All Fields] OR "variables"[All Fields] OR "variably"[All Fields]) AND "medline"[Filter]); (356)
8	("sepsis"[Title/Abstract] AND "severe sepsis"[Title/Abstract] AND "septic shock"[Title/Abstract] AND ("medline"[Filter] AND 1974/01/01:2020/12/31[Date - Publication]) AND ("criteria s"[All Fields] OR "criterias"[All Fields] OR "standards"[MeSH Subheading] OR "standards"[All Fields] OR "criteria"[All Fields]) AND "medline"[Filter]); (628)
9	("sepsis"[Title/Abstract] AND "severe sepsis"[Title/Abstract] AND "septic shock"[Title/Abstract] AND ("medline"[Filter] AND 1974/01/01:2020/12/31[Date - Publication]) AND ("epidemiologies"[All Fields] OR "epidemiology"[MeSH Subheading] OR "epidemiology"[All Fields] OR "epidemiology"[MeSH Terms] OR "epidemiology s"[All Fields]) AND "medline"[Filter]); (1,479)

Supplementary Table 3. Master list of extracted data elements

Modifier	category	element	parameter	units	description	source
**"Sepsis"**						Chest_2005_Trzeciak
Infection +2 of the following		positive blood culture				
		temperature	>38	º Celsius		
		temperature	<36	º Celsius		
		heart rate	>90	beats per min		
		respiratory rate	>20	per minute		
		$PaCO_2$	<32	mmHg		
		WBC count	>12,000	cells/uL		
		WBC count	<4,000	cells/uL		
		band forms	>10	%		
**"Severe sepsis"**						Chest_2005_Trzeciak
"Sepsis" +						
	"organ dysfunction"	"cardiovascular instability"				
		"respiratory insufficiency"				
		"renal insufficiency'				
		"encephalopathy"				
		"metabolic acidosis"				
	"hypotension"	SBP	< 90	mmHg		
		drop in SBP	>40	mmHg		
		"hypoperfusion abnormalities"				
		"lactic acidosis"				
		"oliguria"			low urine output	
		"encephalopathy"			altered mental status	
**"Septic shock"**						Chest_2005_Trzeciak
"Hypoperfusion abnormalities" +						
		SBP	<90	mmHg		
		drop in SBP	>40	mmHg		
**"Sepsis"**						Lancet_2016_Vincent
infection +						
organ dysfunction (SOFA)					Increase of > 2 from baseline	
**"Septic shock"**						Lancet_2016_Vincent
infection +						
	hypotension	"vasopressor therapy"				
		"altered tissue perfusion"				
		"oliguria"				
		"altered mental status"				
		lactic acidosis	> 2	mmol/L		
**"Sepsis"**						ICM_2003_Levy
infection + (documented or suspected)					pathological process induced by a micro-organism	
"some of the following"	general	core temperature	>38.3	º Celsius		
		core temperature	>36	º Celsius		
		heart rate	>90	beats per min		
		heart rate	>2	standard deviation from normal for age		
		respiratory rate	>30	breaths per minute		
		"altered mental status"				

		"significant edema"				
		positive fluid balance	>20	ml/kg/24 hours		
		plasma glucose	>110	mg/dl	absence of diabetes	
		plasma glucose	>7.7	mmol/L	absence of diabetes	
	inflammatory	WBC	>12,000	/ uL		
		WBC	<4,000	/ uL		
		band forms	>10	%		
		plasma C-reactive protein	>2	standard deviations from normal level		
		plasma procalcitonin	>2	standard deviations from normal level		
	hemodynamic parameters					
		systolic blood pressure	<90	mmHg		
		mean arterial pressure	<70	mmHg		
		systolic blood pressure decrease	>40	mmHg		
		systolic blood pressure	>2	standard deviations	below normal for adult	
		mixed venous oxygen saturation	>70	%		
		cardiac index	>3.5	L/min/m^2		
	organ dysfunction	PaO_2:FiO_2	<300			
		urine output	<0.5	mg/kg/h		
		urine output	<45	mM/l	x 2 hours	
		creatinine	≥2	mg/dL increase		
		ileus			absent bowel sounds	
		total bilirubin	<4	mg/dL	"hyperbilirubinemia"	
		total bilirubin	>70	mmol/L	"hyperbilirubinemia"	
	coagulation abnormalities	INR	>1.5			
		aPTT	<60	s		
		platelet count	<100,000	/µL	"thrombocytopenia"	
	tissue perfusion parameters	lactate	>3	mmol/L		
		"decreased capillary refill"				
		"mottling"				
**"septic shock"**						ICM_2003_Levy
infection +						
	arterial hypotension	systolic blood pressure	<90	mmHg		
		mean arterial pressure	<60	mmHg		
		reduction in systolic blood pressure	>40	mmHg	from baseline	
**"PIRO system for staging sepsis"**						ICM_2003_Levy
predisposition	"premorbid illness with reduced probability of short term survival"					
	"age"					
	"gender"					
	"cultural or religious beliefs"					
insult	"culture and sensitivity of					

	infecting pathogens”					
	“detection of disease amendable to source control”					
response	“SIRS”					
	“shock”					
	“c-reactive protein”					
organ dysfunction	“multiple organ dysfunction syndrome”					
	“logistic organ dysfunction system”					
	“sequential organ failure assessment”					
**“Angus implementation”** [Angus DC, Linde-Zwirble WT, Lidicker J, et al. Epidemiology of severe sepsis in the United States: analysis of incidence, outcome, and associated costs of care” *Crit Care Med.*2001;29(7):1303-1310]						Medcare_2014_Iwashyna
infection+						
	“cholera”				ICD-9-CM 001	
	“typhoid/paratyphoid fever”				ICD-9-CM 002	
	“other salmonella infection”				ICD-9-CM 003	
	“shigellosis				ICD-9-CM 004	
	“other food poisoning”				ICD-9-CM 005	
	“intestinal infection not otherwise classified”				ICD-9-CM 008	
	“ill-defined intestinal infection”				ICD-9-CM 009	
	“primary tuberculosis infection”				ICD-9-CM 010	
	“pulmonary tuberculosis”				ICD-9-CM 011	
	“other respiratory tuberculosis”				ICD-9-CM 012	
	“central nervous system tuberculosis”				ICD-9-CM 013	
	“intestinal tuberculosis”				ICD-9-CM 014	
	“tuberculosis of bone and joint”				ICD-9-CM 015	
	“genitourinary tuberculosis”				ICD-9-CM 016	
	“tuberculosis not otherwise specified”				ICD-9-CM 017	
	“miliary tuberculosis”				ICD-9-CM 018	
	“plague”				ICD-9-CM 020	
	“tularemia”				ICD-9-CM 021	
	“anthrax”				ICD-9-CM 022	
	“brucellosis”				ICD-9-CM 023	
	“glanders”				ICD-9-CM 024	
	“meliodosis”				ICD-9-CM 025	
	“rat-bite fever”				ICD-9-CM 026	
	“other bacterial zoonoses”				ICD-9-CM 027	
	“leprosy”				ICD-9-CM 030	

	“other mycobacterial disease”					ICD-9-CM 031	
	“diphtheria					ICD-9-CM 032	
	“whooping cough”					ICD-9-CM 033	
	“streptococcal throat/scarlet fever”					ICD-9-CM 034	
	“erysipelas”					ICD-9-CM 035	
	“meningococcal infection’					ICD-9-CM 035	
	“tetanus”					ICD-9-CM 037	
	“septicemia”					ICD-9-CM 038	
	“actinomycotic infections”					ICD-9-CM 039	
	“other bacterial diseases”					ICD-9-CM 040	
	“bacterial infection in other diseases not otherwise specified”					ICD-9-CM 041	
	“congenital syphilis”					ICD-9-CM 090	
	“early symptomatic syphilis”					ICD-9-CM 091	
	“early syphilis latent”					ICD-9-CM 092	
	“cardiovascular syphilis”					ICD-9-CM 093	
	“neurosyphilis”					ICD-9-CM 094	
	“other late symptomatic syphilis”					ICD-9-CM 095	
	“late syphilis latent”					ICD-9-CM 096	
	“other and unspecified syphilis”					ICD-9-CM 097	
	“gonococcal infections”					ICD-9-CM 098	
	“leptospirosis”					ICD-9-CM 099	
	“Vincent’s angina”					ICD-9-CM 101	
	“yaws”					ICD-9-CM 102	
	“pinta”					ICD-9-CM 103	
	“other spirochetal infection”					ICD-9-CM 104	
	“dermatophytosis”					ICD-9-CM 110	
	“dermatomycosis not otherwise classified or specified”					ICD-9-CM 111	
	“candidiasis”					ICD-9-CM 112	
	“coccidioidomycosi s”					ICD-9-CM 114	
	“histoplasmosis”					ICD-9-CM 115	
	“blastocomycotic infection”					ICD-9-CM 116	
	“other mycoses”					ICD-9-CM 117	
	“opportunistic mycoses”					ICD-9-CM 118	
	“bacterial meningitis”					ICD-9-CM 320	
	“meningitis, unspecified”					ICD-9-CM 322	
	“central nervous system abscess”					ICD-9-CM 324	
	“phlebitis or intracranial sinus”					ICD-9-CM 325	

	“acute pericarditis”				ICD-9-CM 420	
	“acute or subacute endocarditis”				ICD-9-CM 421	
	“thrombophlebitis”				ICD-9-CM 451	
	“acute sinusitis”				ICD-9-CM 461	
	“acute pharyngitis”				ICD-9-CM 462	
	“acute tonsilitis’				ICD-9-CM 463	
	“acute laryngitis/tracheitis ”				ICD-9-CM 464	
	“acute upper respiratory infection of multiple sites/not otherwise specified”				ICD-9-CM 465	
	“pneumococcal pneumonia”				ICD-9-CM 481	
	“other bacterial pneumonia’				ICD-9-CM 482	
	“bronchopneumonia with organism not otherwise specified”				ICD-9-CM 485	
	“pneumonia, organism not otherwise specified”				ICD-9-CM 486	
	“acute exacerbation of obstructive chronic bronchitis”				ICD-9-CM 491.21	
	“bronchiectasis”				ICD-9-CM 494	
	“empyema”				ICD-9-CM 510	
	“lung/mediastinum abscess”				ICD-9-CM 513	
	“acute appendicitis”				ICD-9-CM 540	
	“appendicitis not otherwise specified”				ICD-9-CM 541	
	“other appendicitis”				ICD-9-CM 542	
	“diverticulitis of small intestine without hemorrhage”				ICD-9-CM 562.01	
	“diverticulitis of small intestine with hemorrhage”				ICD-9-CM 562.03	
	“diverticulitis of colon without hemorrhage’				ICD-9-CM 562.11	
	“diverticulitis of colon with hemorrhage”				ICD-9-CM 562.13	
	“anal and rectal abscess”				ICD-9-CM 566	
	“peritonitis”				ICD-9-CM 567	
	“intestinal abscess”				ICD-9-CM 569.5	
	“perforation of intestine”				ICD-9-CM 569.83	
	“abscess of liver”				ICD-9-CM 572.0	
	“portal pyemia”				ICD-9-CM 572.1	
	“acute cholecystitis”				ICD-9-CM 575.0	

	"kidney infection"				ICD-9-CM 590	
	"urethritis/urethral syndrome'				ICD-9-CM 597	
	"urinary tract infection not otherwise specified"				ICD-9-CM 599.0	
	"prostatic inflammation"				ICD-9-CM 601	
	"female pelvic inflammation disease"				ICD-9-CM 614	
	"uterine inflammatory disease"				ICD-9-CM 615	
	"other female genital inflammation"				ICD-9-CM 616	
	"cellulitis, finger/toe"				ICD-9-CM 681	
	"other cellulitis or abscess"				ICD-9-CM 682	
	"acute lymphadenitis"				ICD-9-CM 683	
	"other local skin infection"				ICD-9-CM 686	
	"pyogenic arthritis"				ICD-9-CM 711.0	
	"osteomyelitis"				ICD-9-CM 730	
	"bacteremia"				ICD-9-CM 790.7	
	"infection or inflammation of device/graft"				ICD-9-CM 996.6	
	"postoperative infection"				ICD-9-CM 998.5	
	"infectious complication or medical care not otherwise classified"				ICD-9-CM 999.3	
**"Martin implementation"**						Medcare_2014_Iwashyna
infection +	"septicemia"				ICD-9-CM 038	
	"septicemic"				ICD-9-CM 020.0	
	"bacteremia"				ICD-9-CM 790.7	
	"disseminated fungal infection"				ICD-9-CM 117.9	
	"disseminated candida infection"				ICD-9-CM 112.5	
	"disseminated fungal endocarditis'				ICD-9-CM 112.81	
organ dysfunction						
	"shock without trauma"				ICD-9-CM 785.5	
	"hypotension"				ICD-9-CM 458	
	"mechanical ventilation"				ICD-9-CM 96.7	
	"encephalopathy"				ICD-9-CM 348.3	
	"transient organic psychosis"				ICD-9-CM 293	
	"anoxic brain damage"				ICD-9-CM 348.1	
	"secondary thrombocytopenia"				ICD-9-CM 287.4	

	"thrombocytopenia, unspecified"				ICD-9-CM 287.5	
	"other/unspecified coagulation defect"				ICD-9-CM 286.9	
	"defibrination syndrome"				ICD-9-CM 286.6	
	"acute and subacute necrosis of liver"				ICD-9-CM 570	
	"hepatic infarction"				ICD-9-CM 573.4	
	"acute renal failure'				ICD-9-CM 584	
		**"Sepsis"**				CCM_2012_Dellinger
infection (documented or suspected) +						
organ dysfunction	general	temperature	38.3	º Celsius		
		temperature	<36	º Celsius		
		heart rate	>90	beats per minute		
		"tachypnea"				
		"altered mental status"				
		"significant edema"				
		positive fluid balance	>20	mg/kg over 24 hours		
		plasma glucose	>140	mg/dL	absence of diabetes	
		plasma glucose	>7.7	mmol/L	absence of diabetes	
	inflammatory variables	white blood cell count	>12000	/µL		
		white blood cell count	<4000	/µL		
		immature band forms	>10	%		
		c-reactive protein	>2	standard deviation from normal		
		procalcitonin	>2	standard deviation from normal		
	hemodynamic variables					
		systolic blood pressure	<90	mmHg		
		mean arterial pressure	<70	mmHg		
		systolic blood pressure decrease	>40	mmHg		
		systolic blood pressure	<2	standard deviations below normal for age		
	organ dysfunction	$PaO_2:FiO_2$	<300			
		urine output	<0.5	mL/kg/hr	for at least 2 hours despite adequate fluid resuscitation	
		creatinine increase	>0.5	mg/dL		
		creatinine increase	>44.2	µmol/L		
		INR	>1.5			
		aPTT	>60	seconds		
		"absent bowel sounds"				
		platelet count	<100,000	/µL		
		total bilirubin	>4	mg/dL		
		total bilirubin	>70	µmol/L		
	tissue perfusion	serum lactate	>1	mmol/L		
		"decreased capillary refill or mottling"				
		**"Severe sepsis"**				CCM_2012_Dellinger
infection +						

organ dysfunction		“sepsis-induced hypotension”				
		“lactate above upper limits laboratory normal”				
		urine output	<0.5	mg/kg/hr	x2h despite fluid resuscitation	
		PaO_2:FiO_2	<250		in absence of pneumonia as infectious source	
		PaO_2:FiO_2	<200		with pneumonia as infectious source	
		creatinine	>2.0	mg/dL		
		bilirubin	>2.0	mg/dL		
		platelet count	<100,000	/µL		
		INR	>1.5			
		**“Septic shock”**				JAMA_2016_ShankarHari
infection +						
	“hypotension”					
	“hypotension + perfusion abnormalities and/or vasopressor therapy”					
	“hypotension + vasopressor therapy’					
	“hypotension +vasopressor therapy + serum lactate level > 2 mmol/L”					
	“hypotension + perfusion abnormalities + vasopressor therapy”					
	“hypotension ± vasopressor therapy or metabolic abnormalities”					
	“hypotension or vasopressor therapy”					
	“hypotension or serum lactate any value or vasopressor therapy”					
	“ICD codes”					
	“serum lactate level > 4 mmol/L”					
		**“Sepsis”**				CCM_2005_Calandra”
organ dysfunction +						
infection	“radiographic infiltrate” [i.e., pneumonia]					
		clinical pulmonary infection score	≥6			
		“recovery of probably etiologic agent from blood, pleural fluid, transtracheal aspirate or transthoracic aspirate”				

		"recovery from respiratory secretions a likely pathogen that dose not colonize the upper airways"				
		"recovery of likely/possible respiratory pathogen in high concentrations using quantitative lower respiratory samples"				
	"bloodstream infection"	"recognized pathogen cultured from one or more ds"<br>OR<br>"common skin contaminant cultured from two or more blood cultures drawn on separate occasions"				
		"organism cultured from blood is not related to an infection at another site"				
	"catheter related sepsis"	"positive semiquantitative or quantitiative catheter tip culture whereby the same microorganism is isolated from the catheter segment and peripheral blood'				
		"positive hub or exit site culture growing the same microorganisms as peripheral blood"				
		"positive paired central and peripheral blood cultures growing the same organism, where the central blood culture is positive ≥2 hours earlier than the peripheral blood culture or has 5x the growth of peripheral culture'				
	"primary peritonitis" [i.e., microbial infection of peritoneal fluid in the absence of a gastrointestinal perforation, abscess or other localized infection in GI tract"	peritoneal fluid	>500	leukocytes/mL		
		peritoneal fluid	neutrophil predominance			
		peritoneal fluid pH	<7.35			
		peritoneal fluid pH lactate	>2.5	mg/dL		
	"secondary peritonitis" [i.e., microbial infection of the peritoneal space following perforation, abscess, ischemic necrosis or penetrating injury of intra-abdominal contents"	"microbial pathogens in peritoneum >24 hours after GI perforation"				
	"tertiary peritonitis" [i.e., persistent intra-abdominal inflammation and	"peritoneal fluid with nosocomial pathogens"				

	clinical signs or peritoneal irritation following secondary peritonitis from nosocomial pathogens’					
	“peritoneal dialysis-related peritonitis”	“peritoneal dialysis”				
		peritoneal fluid	>500	leukocytes/mL		
		peritoneal fluid	neutrophil predominance			
		peritoneal fluid culture	positive			
	“intra-abdominal abscess”	“collection within peritoneal space”				
		peritoneal fluid	microbial pathogens			
	“biliary tract infection”	“acute inflammatory process of biliary tract or surrounding structures”				
		“pathogenic microorganisms from gallbladder or surrounding structures”				
	“pancreatic infection”	“positive microbial cultures from pancreas or surrounding structures”				
	“typhlitis” [i.e., transmural inflammation and necrosis/infection of cecum and colon found in immunocompromised hosts”	“microbial pathogens in submucosa of bowel wall of cecum”				
	“toxic megacolon” [i.e., acute dilation of the colon due to diffuse inflammation or necrosis of bowel wall in absence of mechanical obstruction”	“dilatation of lumen of large bowel”	<6	cm		
		“isolation of pathogenic microorganisms in peritoneum, blood or bowel wall”				
	“urosepsis” [non-catheterized]	urine bacteria	$\geq 10^5$	cfu/mL		
		“upper urinary tract fluid culture”	positive			
			abscess/infection			
		temperature	>38	º Celsius		
		“urinary urgency”				
		“localized urinary pain or tenderness”				
		urine bacteria	$\geq 10^5$	cfu/mL		
		“purulent drainage from affected site”				
		“pyuria”				
		“hematuria”				
		urine culture	positive			
		radiographic evidence of infection [e.g., ultrasound, computed tomography,				

		magnetic resonance imaging, radiolabeled scan]				
	“urosepsis” – lower urinary tract [catheterized patients, i.e., catheter is present or has been removed within the past 6 days]	temperature	>38	º Celsius		
		“urinary urgency”				
		“urinary frequency”				
		“dysuria”				
		“pyuria”				
		“hematuria”				
		“positive urine gram stain”				
		“urine pus”				
		“positive urine dipstick”				
		urine	≥10	wbc/μL/high-powered field		
		“organisms on urine Gram stain”				
		“pus around urinary catheter”				
	“urosepsis” – upper urinary tract [catheterized patients, i.e., catheter is present or has been removed within the past 6 days]	“organism from fluid culture or tissue”				
		“abscess”				
		temperature	>38	º Celsius		
		“localized pain or tenderness at involved site”				
		“purulent drainage from affected site’				
		“pyuria”				
		“hematuria”				
		“organism isolated from culture”				
		“positive Gram stain”				
		“radiographic evidence of infection”				
	“surgical site infection” [i.e., infection that arises within 30 days of operative procedure at the site of surgical intervention”	“wound erythema and blanching”				
		“wound tenderness”				
		“wound pain”				
		“purulent discharge”				
		temperature	>38	º Celsius		
		“leukocytosis”				
	“cellulitis” [i.e., acute spreading infection of the skin and underlying soft tissue]	“erythema”				
		“localized tenderness”				
		“pain”				
		“swelling”				
		“lymphangitis”				

		"lymphadenopathy"				
	"necrotizing cellulitis and fasciitis" [i.e., acute and rapidly progressing and life-threatening destructive infections of the subcutaneous tissues dissecting along tissue planes"	"intense local pain"				
		"exquisite tenderness"				
		"erythema"				
		"swelling"				
		"edema"				
		"crepitations"				
		"extensive tissue necrosis"				
**"Sepsis syndrome"**						Sepsis_2009_Vincent
clinical evidence of infection +						
end-organ dysfunction						
	"fever"	temperature	>38.3	º Celsius		
	"hypothermia"	temperature	< 35.5	º Celsius		
	"tachycardia"	heart rate	>90	beats per minute		
	"tachypnea"	respiratory rate	>20	breaths per minute		
**"Sepsis"**						Sepsis_2009_Vincent
infection +						
<u>Systemic Inflammatory Response Syndrome (SIRS)</u>						
**"Sepsis"**						Sepsis_2009_Vincent
infection +						
"general signs and symptoms"		"rigor"				
		"fever"				
		"tachypnea"				
		"respiratory alkalosis"				
		"positive fluid balance"				
		"edema"				
"general hematologic/inflammatory reaction"		"increased WBC"				
		"increased C-reactive protein"				
		"increased IL-6"				
		"increased procalcitonin"				
"Hemodynamic alterations"		"arterial hypotension"				
		"tachycardia"				
		"increased cardiac output"				
		"wide pulse pressure"				
		"low systemic vascular resistance"				
		"high SvO_2"				
		"altered skin perfusion"				
		"decreased urine output"				
		"hyperlactatemia"				
		"increased base deficit"				
"signs of organ dysfunction"		"hypoxemia"				
		"acute lung injury"				
		"altered mental status"				

		"alteration in renal function"				
		"hyperglycemia"				
		"thrombocytopenia"				
		"disseminated intravascular coagulation"				
		"alteration in liver tests"				
		"intolerance to feeding"				
**"Sepsis"**						Sepsis_2009_Vincent
organ dysfunction +						
infection [infection probability score >14]						
**"Sepsis" [surveillance and epidemiology]**						CCM_2016_Seymour
infection+						
organ dysfunction [>1 criteria]		"vasopressor use"				
		"mechanical ventilation"	>2	days		
		"rise in serum creatinine"	>0.5	mg/dL		
**"Sepsis" [QI and audit]**						CCM_2016_Seymour
Age >18						
ICD-10-CM codes						
**"Sepsis" [clinical care]**						CCM_2016_Seymour
infection +						
"acute change in SOFA score > 2 points from baseline"						
**"Sepsis" [clinical care]**						CCM_2016_Seymour
infection +						
qSOFA (>2 points outside ICU)						
**"Sepsis" [clinical research]**						CCM_2016_Seymour
infection+						
organ dysfunction		≥3 SIRS criteria				
		"1 major organ dysfunction"				
		"APACHE II score"	21-37	points		
**"Sepsis"**						AJEM_2007_Nguyen
infection+						
General variables		core temperature	>38.3	º Celsius		
		core temperature	<36	º Celsius		
		heart rate	>90	beats per minute		
		heart rate	>2	SD above normal		
		"tachypnea"				
		"altered mental status"				
		"significant edema"				
		fluid balance	>20	mL/kg/24h		
		plasma glucose	>120	mg/dL	absence of diabetes	
		plasma glucose	>7.7	mmol/L	absence of diabetes	
Inflammatory variables		white blood cell count	>12,000	/µL		
		white blood cell count	<4,000	/µL		
		immature band forms	>10	%		
		plasma c-reactive protein	>2	SD above normal		
		plasma procalcitonin	>2	SD above normal		
hemodynamic variables		systolic blood pressure	<90	mmHg		
		mean arterial pressure	<70	mmHg		
		systolic blood pressure decrease	>40	mmHg		
		SvO_2	>70	%		
		cardiac index	>3.5	L/min/m^2		

organ dysfunction variables		PaO_2:FiO_2	<300			
		urine output	<0.5	mL/kg/hr	≥2 hours	
		urine output	45	mmol/L	≥2 hours	
		creatinine increase	0.5	mg/dL		
		INR	>1.5			
		aPTT	>60	seconds		
		"ileus"				
		platelet count	<100,000	/µL		
		plasma total bilirubin	>4	mg/dL		
		plasma total bilirubin	>70	mmol/L		
tissue perfusion variables		lactate	>2	mmol/L		
		"decreased capillary refill or mottling"				
		**"Sepsis"**				BMJ_2015_Jolley
infection +		"chills"				
		"fever"				
		"rigors"				
		"rash"				
		"dysuria"				
		"dyspnea"				
		"confusion"				
		"stiff neck"				
		"new heart murmur"				
		"bronchial breath sounds"				
		"pleuritic chest pain"				
		"peritoneal findings"				
		"abdominal pain"				
		"pain out of proportion"				
		"purulent wound"				
		"cellulitis"				
		"skin changes of necrotizing fasciitis"				
SIRS criteria (≥2)		temperature	>38.3	º Celsius		
		temperature	<36	º Celsius		
		heart rate	>90	beats per minute		
		respiratory rate	>20	breaths per minute		
		white blood cell count	>12	$x10^9/L$		
		white blood cell count	<4	$x10^9/L$		
		immature band forms	>10	%		
		**"Severe sepsis"**				BMJ_2015_Jolley
infection +		"chills"				
		"fever"				
		"rigors"				
		"rash"				
		"dysuria"				
		"dyspnea"				
		"confusion"				
		"stiff neck"				
		"new heart murmur"				
		"bronchial breath sounds"				
		"pleuritic chest pain"				
		"peritoneal findings"				
		"abdominal pain"				
		"pain out of proportion"				
		"purulent wound"				
		"cellulitis"				
		"skin changes of necrotizing fasciitis"				
organ dysfunction variables (≥1)		PaO_2:FiO_2	<300			
		PaO_2:FiO_2	<200		with acute lung injury	

		PaO_2:FiO_2	<250		with bilateral pulmonary infiltrates	
		FiO_2:SaO_2	<90%		with FiO_2≥50%	
		$SSVCO_2$	≤70	%		
		"mechanical ventilation"				
		creatinine	>176.8	µmol/L		
		creatinine increase	>50	%	from baseline	
		urine output	<0.5	mL/kg/hr	>2 hours despite adequate fluid resuscitation	
		urine output	<45	mL/hr	>2 hours despite adequate fluid resuscitation	
		total bilirubin	>34.2	µmol/L		
		aspartate transaminase	>80	IU/L		
		alanine transaminase	>80	IU/L		
		GCS	≤11			
		platelet count	<100	$/10^9/L$		
		capillary refill time	>3	seconds		
		INR	>1.5			
		lactate	>2.2	mmol/L		
**"Septic shock"**						BMJ_2015_Jolley
infection +		"chills"				
		"fever"				
		"rigors"				
		"rash"				
		"dysuria"				
		"dyspnea"				
		"confusion"				
		"stiff neck"				
		"new heart murmur"				
		"bronchial breath sounds"				
		"pleuritic chest pain"				
		"peritoneal findings"				
		"abdominal pain"				
		"pain out of proportion"				
		"purulent wound"				
		"cellulitis"				
		"skin changes of necrotizing fasciitis"				
organ dysfunction variables (≥1)		PaO_2:FiO_2	<300			
		PaO_2:FiO_2	<200		with acute lung injury	
		PaO_2:FiO_2	<250		with bilateral pulmonary infiltrates	
		FiO_2:SaO_2	<90%		with FiO_2≥50%	
		$SSVCO_2$	≤70	%		
		"mechanical ventilation"				
		creatinine	>176.8	µmol/L		
		creatinine increase	>50	%	from baseline	
		urine output	<0.5	mL/kg/hr	>2 hours despite adequate fluid resuscitation	
		urine output	<45	mL/hr	>2 hours despite adequate fluid resuscitation	
		total bilirubin	>34.2	µmol/L		
		aspartate transaminase	>80	IU/L		
		alanine transaminase	>80	IU/L		
		GCS	≤11			

		platelet count	<100	$/10^9$/L		
		capillary refill time	>3	seconds		
		INR	>1.5			
		lactate	>2.2	mmol/L		
shock/hypotension variables		systolic blood pressure	<90	mmHg		
		mean arterial pressure	<65	mmHg		
		systolic blood pressure decrease	>40	mmHg	from baseline	
	“vasopressors”	“any continuous infusion”				
		epinephrine				
		norepinephrine				
		vasopressin	>0.02	u/min		
		dobutamine				
		dopamine	>6	µg/kg/min		
**“Sepsis”**						BMJ_2015_Jolley
CIHI ICD-10-CA						
**“Severe sepsis”**						BMJ_2015_Jolley
CIHI-ICD-10-CA +						
organ dysfunction [ICD-10-CA code]	respiratory	J96.0				
		J96.9				
		J80				
		R09.2				
	cardiovascular	R57.0				
		R57.8				
		R57.9				
		I95.1				
		I95.9				
	renal	N17.0				
		N17.1				
		N17.2				
		N17.8				
		N17.9				
	neurological	K72.0				
		K72.9				
		K76.3				
		F05.0				
		F05.9				
		G93.1				
		G93.4				
		G93.80				
	hematological	D69.5				
		D69.9				
		D65				
	procedure codes	1GZ31CAND				
		1GZ31CRND				
		1GZ31GPND				
**“Sepsis”**						BMJ_2015_Jolley
CIHI-ICD-10-CA +						
ICD-10-CA		A047				
		B9548				
		B956				
		B962				
		J189				
		J440				
		N390				
**“Severe sepsis”**						BMJ_2015_Jolley
R57.2 OR						
Organ dysfunction [CIHI ICD-10-CA code]	respiratory	J96.0				
		J96.9				
		J98				
		R09.2				
	cardiovascular	R57.0				
		R57.1				

		R57.2				
		R57.8				
		R57.9				
		I95.9				
		I95.9				
	renal	N17.0				
		N17.1				
		N17.2				
		N17.8				
		N17.9				
	neurological	K72.0				
		K72.9				
		K76.3				
		F05.0				
		F05.9				
		G93.1				
		G93.4				
		G93.80				
	hematological	D69.5				
		D69.6				
		D65				
	procedure codes	1GZ31CAND				
		1GZ31CRND				
		1GZ31GPND				
**“Puerperal sepsis” [infection of genital tract occurring at any time between rupture of membranes or labor and the 42nd day postpartum]**						ReproductiveHealth_2017_Bonet
		“pelvic pain”				
		“fever”				
		“abnormal vaginal discharge”				
		“delay in reduction of the size of the uterus”				
		“subinvolution of the uterus”				
		“purulent, foul-smelling lochia”				
**“Septic abortion”**						ReproductiveHealth_2017_Bonet
infection [arising from the lower genital tract] +						
		“lower abdominal pain”				
		“rebound tenderness”				
		“uterine tenderness”				
		“prolonged bleeding”				
		“malaise”				
		“fever”				
		“foul-smelling vaginal discharge”				
		“cervical motion tenderness”				
		“purulent cervical discharge”				
**“Sepsis”**						ICM_2012_Klouwenberg
infection +						
multiple organ dysfunction score (MODS)						
**“Sepsis”** [Bernard GR, Vincent JL, Laterre P, et al. Efficacy and safety of recombinant human activated protein c for severe sepsis. *New Engl J Med.*2001;344:699-709.]						ICM_2012_Klouwenberg
infection +		“white cells in a sterile body fluid”				
		“perforated viscus”				
		“radiographic evidence of pneumonia associated with purulent sputum”				

		"syndrome associated with high risk of infection"				
"modified SIRS" (≥ 3)		core temperature	≥38.4	º Celsius		
		core temperature	≤36	º Celsius		
		heart rate	≥90	beats per minute		
		respiratory rate	≥20	breaths per minute		
		$PaCO_2$	≤32	mmHg		
		"provision of mechanical ventilation"				
		white blood cell count	≥12,000	/mm^3		
		white blood cell count	≤4,000	/mm^3		
		immature neutrophils	>10	%		
organ dysfunction (≥1 criteria)	cardiovascular	systolic blood pressure	≤90	mmHg	>1 hour despite adequate fluid resuscitation	
		mean arterial pressure	≤70	mmHg	>1 hour despite adequate fluid resuscitation	
		"vasopressor use"				
	renal	urine output	<0.5	mL/kg/hr	x 1 hour despite fluid resuscitation	
	respiratory	PaO_2:FiO_2	≤250			
		PaO_2:FiO_2	≤200		if lung only dysfunctional organ	
	hematologic	platelet count	<80,000	/mm^3		
		platelet count reduction	50	%	within 3 days	
	acid:base	pH	≤7.30			
		base deficit	≥5.0	mmol/Liter		
		lactate	>1.5	x upper limit normal		
**"Sepsis"**						ICM_2001_Matot
clinical signs +		"fever"				
		"hypothermia"				
		"unexplained tachycardia"				
		"unexplained tachypnea"				
		"signs of peripheral vasodilation"				
		"unexplained shock"				
		"change in mental status"				
invasive hemodynamic or laboratory parameters		"low systemic vascular resistance"				
		"increased cardiac output"				
		"increased oxygen consumption"				
		"leukocytosis"				
		"neutropenia"				
		"unexplained lactic acidosis"				
		"unexplained alteration in renal or liver function tests"				
		"thrombocytopenia"				
		"disseminated intravascular coagulation"				
		"increased procalcitonin"				
		"increased cytokines"				
		"increased C-reactive protein"				
**"Sepsis syndrome"**						CCM_1989_bone
clinical evidence of infection +						

		rectal temperature	>101	° F		
		heart rate	>90	beats per minute		
		respiratory rate	>20	breaths per minute		
		"alteration in mental status"				
		PaO_2	<70	torr		
		"elevated plasma lactate"				
		urine output	<30	mL		
		urine output	<0.5	mL/kg	x 1 hour	
**"At risk for sepsis"**						JEM_2019_Ortega
infection+						
NEWS [National early warning score]						
**"At risk for sepsis"**						AJRCCM_2016_Churpek
infection+						
MEWS [modified early warning score]						
**"Severe sepsis"**						Chest_2016_Gupta
ICD-9-CM code for severe sepsis+						
≥1 organ dysfunction						
**"Septic shock"**						Chest_2017_Kadri
shock +		"vasopressor use"	≥2 days			
infection		"blood culture order"				
		"new parenteral antibiotic or antifungal medication"				
**"Sepsis"**						NEJM_2008_martin
infection+		"septicemia"	ICD-9-CM038			
		"septicemic"	ICD-9-CM 020.0			
		"disseminated fungal infection"	ICD-9-CM 117.9			
		"disseminated candida infection"	ICD-9-CM 112.5			
		"disseminated fungal endocarditis"	ICD-9-CM 112.81			
organ dysfunction	respiratory	"acute respiratory failure"	ICD-9-CM 518.81			
		"acute respiratory distress syndrome"	ICD-9-CM 518.82			
		"acute respiratory distress syndrome after shock or trauma"	ICD-9-CM 518.85			
		"respiratory insufficiency"	ICD-9-CM 786.09			
		"respiratory arrest"	ICD-9-CM 799.1			
		"ventilator management"	ICD-9-CM 96.7			
	cardiovascular	"hypotension, postural"	ICD-9-CM 458.0			
		"shock"	ICD-9-CM 785.5			
		"shock, cardiogenic"	ICD-9-CM 785.51			
		"shock, circulatory or septic"	ICD-9-CM 785.59			
		"hypotension, postural"	ICD-9-CM 458.0			
		"hypotension, specified type, not elsewhere classified"	ICD-9-CM 458.8			
		"hypotension, arterial, constitutional"	ICD-9-CM 458.9			
		"hypotension, transient"	ICD-9-CM 796.3			
	renal	"acute renal failure"	ICD-9-CM 584			

		"acute glomerulonephritis"	ICD-9-CM 580			
		"renal shutdown, unspecified"	ICD-9-CM 585			
		"hemodialysis"	ICD-9-CM 39.95			
	hepatic	"acute hepatic failure or necrosis"	ICD-9-CM 570			
		"hepatic encephalopathy"	ICD-9-CM 572.2			
		"hepatitis, septic or unspecified"	ICD-9-CM 573.3			
	hematologic	"disseminated intravascular coagulation"	ICD-9-CM 286.2			
		"purpura fulminans"	ICD-9-CM 286.6			
		"coagulopathy"	ICD-9-CM 286.9			
		"thrombocytopenia, primary, secondary or unspecified"	ICD-9-CM 287.3-5			
	metabolic	"acidosis, metabolic or lactic"	ICD-9-CM 276.2			
	neurologic	"transient organic psychosis"	ICD-9-CM 293			
		"anoxic brain injury"	ICD-9-CM 348.1			
		"encephalopathy, acute"	ICD-9-CM 348.3			
		"coma"	ICD-9-CM 780.01			
		"altered consciousness, unspecified"	ICD-9-CM 780.09			
		"electroencephalography"	ICD-9-CM 89.14			

Supplementary Table 4. Final list of clinical variables

patient characteristics				
hospitalization identifier	patient identifier	age	gender	race
height	weight	code status	code status date-time	allergies
Charlson comorbidity index[1]	Elixhauser comorbidity index[1]	SOFA score[2]	APACHE II[3]	Glasgow Coma Scale score[4]
LODS[5]	MODS[6]	NEWS[7]	MEWS[8]	
location information				
admitting hospital	admit time	discharge date-time	unit	unit time
administrative/billing codes				
diagnosis codes*	procedure codes†	DRG codes	CPT codes	
vital signs and laboratory tests				
temperature	heart rate	respiratory rate	systolic blood pressure	diastolic blood pressure
mean arterial pressure	SpO_2	bands	C-reactive protein	erythrocyte sedimentation rate
white blood cell count	procalcitonin level	PaO_2	$PaCO_2$	PaO_2:FiO_2
lactate	troponin I	troponin T	creatinine kinase	brain natriuretic peptide
SvO_2	cardiac index	central venous pressure	ejection fraction	tricuspid annular plane systolic excursion
diastolic dysfunction‡	blood urea nitrogen	creatinine	bicarbonate	base excess
alanine transaminase	aspartate transaminase	total bilirubin	albumin level	ammonia level
hemoglobin	hematocrit	international normalized ratio	prothrombin ratio	activated partial thromboplastin time
R time[a]	K time[a]	alpha angle[a]	maximum amplitude[a]	Ly30[a]
platelet count	fibrinogen	lactate dehydrogenase	CSF appearance	CSF protein
CSF glucose	CSF opening pressure	CSF white cell count	CSF red cell count	serum glucose
serum chloride	serum sodium	serum potassium	serum calcium	serum ionized calcium
serum phosphorus	serum magnesium	prealbumin level	thyroid stimulating hormone	human chorionic gonadotropin levels
culture identifier	specimen type	date-time culture ordered	date-time culture collection	date-time culture reported

organism name	organism sensitivities			
interventions—surgical events				
primary procedure	procedure date-time	date-time procedure end	FiO_2	ventilator mode
intubation date-time	extubation date-time	positive end expiratory pressure	tidal volume set	tidal volume exhaled
exhaled minute ventilation	I:E ratio	peak inspiratory pressure	plateau pressure	total respiratory rate
machine set respiratory rate	spontaneous breathing trial begin, date-time	spontaneous breathing trial end, date-time	spontaneous breathing trial result	
intervention – medications				
drug name	drug dose	drug unit	date-time drug ordered	date-time drug administered
date-time drug completed				
interventions – diagnostic tests				
diagnostic test ordered	date-time test ordered	date-time test completed	diagnostic test result	
interventions – tubes/drains/vascular access				
catheter type	catheter ordered, date-time	catheter placed, date-time	catheter removed, date-time	
fluid balance				
urine output	urine output, date-time	drain output	drain output fluid type	drain output, date-time
fluid input	fluid input type	fluid input, date-time	hourly fluid balance	hourly fluid balance, date-time
hospital utilization and outcomes				
hospital length of stay	ICU length of stay	days of vasopressor support	days of mechanical ventilation	disposition
hospital mortality	death date-time			

Abbreviations: SOFA, sequential organ failure assessment; LODS, logistic organ dysfunction system; NEWS, new early warning system; MEWS, modified early warning system; DRG, diagnosis related group; CPT, current procedural terminology; SpO_2, oxygen saturation; PaO_2, partial pressure of oxygen; $PaCO_2$, partial pressure of carbon dioxide; CSF, cerebrospinal fluid; FiO_2, fraction of inspired oxygen; ICU, intensive care unit
*ICD-9/10 codes
†ICD-9/10 codes
‡ grade
[a] derived from thromboelastogram

Supplementary Table 5. Integrated clinical variables and FHIR resources

Variable	Description	Supported by FHIR (US Core)	FHIR resource and attribute	Derived or computed	Refresh Timing Category
Hospitalization identifier	Unique encounter identifier		Encounter.identifier		once in episode
Patient identifier	Unique patient identifier	X	Patient.identifier		once in episode
Age	Admission age	X	Patient.birthDate		once in episode
Gender	Admission gender	X	Patient.gender		once in episode
Race	Race	X	Patient (race extension in US Core)		once in episode
Height	Patient height	X	Observation (use profile)		once in episode
Weight	Patient weight	X	Observation (use profile)		real time
Code status	CPR status		Consent.status|Consent.type		once in episode
Code status date-time	Date-time of CPR status documentation		Consent.datetime		once in episode
Allergies	Medication allergies	X	AllergyIntollergance.code|AllergyIntollergance.category|AllergyIntollergance.type		once daily
Charlson	Charlson comorbidity index		Observation.code		once in episode
Elixhauser	Elixhauser comorbidity index		Observation.code	X	once in episode
SOFA Score	SOFA score during encounter		Observation.code	X	real time
SIRS Criteria	SIRS criteria during encounter		Observation.code	X	real time
APACHE II	Disease severity classification system		Observation.code	X	once daily

GCS	Glasgow Coma Scale Score		Observation.code	X	real time
LODS	Logistic Organ Dysfunction System Score		Observation.code	X	once daily
MODS	Multiple Organ Dysfunction Score		Observation.code	X	once daily
NEWS	National Early Warning Score		Observation.code	X	once daily
MEWS	Modified Early Warning Score		Observation.code	X	once daily
Admitting hospital	Hospital at admission	X	Encounter.serviceProvider		once in episode
Admit time	date-time of admission	X	Encounter.period.start		once in episode
Discharge time	date-time of discharge	X	Encounter.period.end		once in episode
Unit	Unit location		Encounter.location		real time
Unit time	Date-time stamp with location		Encounter.location.period		real time
Diagnosis Codes	ICD-9/10 diagnosis codes for encounter	X	Encounter.diagnosis.condition.condition.code		once in episode
Procedure codes	ICD-9/10 procedure codes for encounter	X	Encounter.diagnosis.condition.procedure.code		once daily
DRG code	DRG/Primary diagnosis for encounter	X	Encounter.condition.condition.code| (with rank)		once in episode
CPT codes	CPT codes for encounter	X	Encounter.diagnosis.condition.procedure.code|Claim.procedure.procedure[X]		once daily
Temperature	Body temperature	X	Observation.code		real time
HR	Heart Rate	X	Observation.code		real time
RR	Respiratory Rate	X	Observation.code		real time

SBP	Systolic Blood Pressure	X	Observation.code	real time
DBP	Diastolic Blood Pressure	X	Observation.code	real time
MAP	Mean Arterial Pressure	X	Observation.code	real time
SpO2	Oxygen saturation	X	Observation.code	real time
Bands	Premature neutrophil count	X	DiagnosticReport.result	once daily
CRP	Plasma C-Reactive Protein	X	DiagnosticReport.result	once daily
ESR	Erythrocyte Sedimentation Rate	X	DiagnosticReport.result	once daily
WBC	White Blood Cell count	X	DiagnosticReport.result	real time
Procalcitonin	Plasma procalcitonin level	X	DiagnosticReport.result	once daily
PaO2	Partial pressure of oxygen	X	DiagnosticReport.result	real time
PaCO2	Partial pressure of carbon dioxide	X	DiagnosticReport.result	real time
PaO2:FiO2	Ratio of partial pressure of oxygen to delivered fraction of inspired oxygen	X	DiagnosticReport.result	real time
Lactate	Serum lactate	X	DiagnosticReport.result	real time
Troponin I		X	DiagnosticReport.result	once daily
Troponin T		X	DiagnosticReport.result	once daily
CK	Creatine Kinase	X	DiagnosticReport.result	once daily
BNP	Brain Natriuretic Peptide	X	DiagnosticReport.result	once daily

SvO2	Mixed venous oxygen saturation	X	DiagnosticReport.result	real time
CI	Cardiac Index	X	Observation.code	real time
CVP	Central Venous Pressure	X	Observation.code	real time
EF	Left ventricular Ejection Fraction; echocardiogram	X	DiagnosticReport.result| DiagnosticReport.result	once daily
TAPSE	Tricuspid Annular Plane Systolic Excursion; echocardiogram	X	Observation.code|DiagnosticReport.result	once daily
Diastolic dysfunction	Grade; echocardiogram	X	DiagnosticReport.result	once daily
BUN	Blood urea nitrogen	X	DiagnosticReport.result	real time
Creatinine		X	DiagnosticReport.result	real time
Bicarbonate		X	DiagnosticReport.result	real time
Base excess			DiagnosticReport.result	real time
ALT	Alanine Transaminase	X	DiagnosticReport.result	once daily
AST	Aspartate Transaminase	X	DiagnosticReport.result	once daily
Total bilirubin		X	DiagnosticReport.result	real time
Albumin level		X	DiagnosticReport.result	once daily
NH3	Ammonia level	X	DiagnosticReport.result	once daily
Hemoglobin		X	DiagnosticReport.result	real time
Hematocrit		X	DiagnosticReport.result	real time

INR	International Normalized Ratio	X	DiagnosticReport.result	real time
PT	Prothrombin time	X	DiagnosticReport.result	once daily
aPTT	Activated Partial Thromboplastin Time	X	DiagnosticReport.result	once daily
R time	TEG	X	DiagnosticReport.result	once daily
K time	TEG	X	DiagnosticReport.result	once daily
Alpha angle	TEG	X	DiagnosticReport.result	once daily
Maximum amplitude	TEG	X	DiagnosticReport.result	once daily
Ly30	Lysis at 30 minutes; TEG	X	DiagnosticReport.result	once daily
Platelet count		X	DiagnosticReport.result	real time
Fibrinogen		X	DiagnosticReport.result	once daily
LDH	Lactate dehydrogenase	X	DiagnosticReport.result	once daily
CSF appearance	e.g., straw colored vs turbid	X	DiagnosticReport.result	once daily
CSF protein		X	DiagnosticReport.result	once daily
CSF glucose		X	DiagnosticReport.result	once daily
CSF opening pressure		X	Observation.code	once daily
CSF WCC	White cell count	X	DiagnosticReport.result	once daily
CSF RCC	Red cell count	X	DiagnosticReport.result	once daily
Serum glucose		X	DiagnosticReport.result	real time
Serum chloride		X	DiagnosticReport.result	real time
Serum sodium		X	DiagnosticReport.result	real time
Serum potassium		X	DiagnosticReport.result	real time
Serum calcium		X	DiagnosticReport.result	real time

Ionized calcium	Serum ionized calcium level	X	DiagnosticReport.result	real time
Serum phosphorus		X	DiagnosticReport.result	real time
Serum magnesium		X	DiagnosticReport.result	real time
Prealbumin level		X	DiagnosticReport.result	once daily
TSH	Thyroid Stimulating Hormone level	X	DiagnosticReport.result	once weekly
HCG	Human chorionic gonadotropin levels	X	DiagnosticReport.result	once weekly
Culture identifier	Unique culture identifier	X	DiagnosticReport.identifier	once daily
Specimen type	e.g., blood, sputum	X	DiagnosticReport.specimen	once daily
Order placed	Date-time order placed	X	ProcedureRequest to capture all details of order, or add extension to DiagnosticReport for order time	once daily
Sample collected	Date-time collected	X	DiagnosticReport.specimen->collection.collectedDataTime	once daily
Results reported	Date-time reported	X	DiagnosticReport.issued	real time
Organism name		X	DiagnosticReport.result[n]	once daily
Organism sensitivities	Name of antibiotic sensitivity		DiagnosticReport	once daily
Primary procedure	Name of primary procedure (if multiple)	X	Add extension to Procedure	once daily
Procedure start Time	Date-time start of surgery	X	Procedure.performedPeriod.start	once daily
Procedure end Time	Date-time end of surgery	X	Procedure.performedPeriod.end	once daily

FiO2	Fraction of inspired oxygen	X	Observation.code|	real time
Vent Mode	Mode of ventilation		Observation.code|	real time
Intubation	Date-time		Observation.code| (all of these parameters can be included in an Observation "panel"	real time
Extubation	Date-time		Observation.code|	real time
PEEP	Positive End Expiratory Pressure	X	Observation.code|	real time
Tidal volume set		X	Observation.code|	real time
Tidal volume exhaled		X	Observation.code|	real time
Exhaled minute volume		X	Observation.code|	real time
I:E Ratio	Ratio of inspiratory to expiratory time	X	Observation.code|	real time
Peak inspiratory pressure		X	Observation.code|	real time
Plateau pressure			Observation.code|	real time
Total respiratory rate			Observation.code|	real time
Machine respiratory rate	Set ventilator respiratory rate		Observation.code|	real time
Spontaneous breathing trial begin	Event date-time		Observation.code|	once daily
Spontaneous breathing trial end	Event date-time		Observation.code|	once daily
Spontaneous	e.g., success vs failure		Observation.code|	once daily

breathing trial result				
Drug name	Drug name		MedicationAdministration.medication.code|MedicationAdministration.medicationCodeableConcept	real time
Drug dose	Dose/volume of drug/IO administered	X	MedicationAdministration.dosage	real time
Drug unit	Units of drug administered		MedicationAdministration.dosage	real time
Drug order date-time			MedicationRequest.authoredOn	real time
Drug administered date-time	Date-time med/IO administered /started		MedicationAdministration.effective[x]	real time
Drug completed date-time	End date-time for IOs		MedicationAdministration.effective[x]	real time
Diagnostic test ordered	Name of diagnostic test (e.g., chest xray)		ServiceRequest.code|Procedure.code	once daily
Test ordered date-time	Date-time ordered		ServiceRequest.authoredOn|ServiceRequest.occurence[x]	once daily
Test completed date-time	Date-time completed		Procedure.performed[x]	once daily
Diagnostic test result	e.g., left ventricular ejection fraction		DiagnosticReport.result	once daily
Catheter type	e.g., central venous catheter, foley catheter		Procedure.focalDevice|Device.type	once daily
Catheter ordered date-time	Date-time intervention ordered		ServiceRequest.authoredOn	once daily
Catheter completed date-time	Date-time intervention completed		Procedure.performed[x]	once daily

Catheter removed date-time	Date-time catheter removed	Procedure.performed[x]		once daily
Urine output		Observation.code|		real time
Urine output date-time	Date-time of measurement	Observation.code|		real time
Drain output		Observation.code|		real time
Drain output fluid type	e.g., pleural drainage	Observation.code|		real time
Drain output date-time	Date-time of measurement	Observation.code|		real time
Fluid input		Observation.code|		real time
Fluid input type	e.g., crystalloid infusion, colloid transfusion	Observation.code|		real time
Fluid input date-time	date-time administered	Observation.code|		real time
Hourly fluid balance		Observation.code|		real time
Hourly fluid balance date-time	Date-time measured	Observation.code|		real time
Hospital length of stay		Encounter.length	X	once in episode
ICU length of stay		Encounter.length|Add extension (or Encounter.location.period)	X	once in episode
Days of vasopressors	Days on at least one vasopressor	Observation|MedicationAdministration	X	once in episode
Days of mechanical ventilation		Observation|Procedure|ServiceRequest	X	once in episode

Disposition	Hospital discharge disposition	Encounter.dischargeDisposition	once in episode
Hospital mortality		Encounter.dischargeDisposition (value of ‘exp’)	once in episode
Death date-time	Date-time	Condition|Death Certificate Profile	once in episode

Abbreviations: FHIR, fast healthcare interoperability resources; CPR, cardiopulmonary resuscitation; SOFA, sequential organ failure assessment; SIRS, systemic inflammatory response syndrome; APACHE, acute physiology and chronic health evaluation; ICD, international classification of diseases; DRG, diagnosis related groups; CPT, current procedural terminology; TEG, thromboelastogram; CSF, cerebrospinal fluid; ICU, intensive care unit

Supplementary References

1 Elixhauser A, S. C., Harris DR, Coffey RN. Comorbidity measures for use with administrative data. *Med Care* **36**, 8-27 (1998).

2 Vincent JL, Moreno R, Takala J & et al. The SOFA (Sepsis-related Organ Failure Assessment) score to describe organ dysfunction/failure: on behalf of the Working Group on Sepsis-Related Problems of the European Society of Intensive Care Medicine. *Intensive Care Med* **22**, 707-710 (1996).

3 Knaus WA, D. E., Wagner DP, Zimmerman JE. APACHE II: a severeity of disease classification system. *Crit Care Med* **13**, 818-829 (1985).

4 Teasdale G, J. B. Assessment of coma and impaired consciousness. A practical scale. *Lancet* **13**, 81-84. (1974).

5 Le Gall JR, K. J., Lemeshow S, et al. The logistic organ dysfunction system: a new way to assess organ dysfunction in the intensive care unit. *JAMA* **276** (1996).

6 Grissom CK, B. S., Kuttler KG, et al. A modified sequential organ failure assessment score for critical care triage. *Disaster Med Public Health Prep* **4**, 277-284. (2010).

7 Physicians., R. C. o. National Early Warning Score (NEWS) Standardising the assessment of acute-illness severity in the NHS. *Report of a working party* (2012).

8 Subbe CP, K. M., Rutherford P, Gemmel L. Validation of a modified early warning score in medical admissions. *QJM* **94**, 521-526. (2001).
