# Supplementary material for: Developing a shared sepsis data infrastructure: a systematic review and concept map to FHIR
Source: NPJ Digit Med. 2022 Apr 4;5:44. doi: 10.1038/s41746-022-00580-2 (PMC8979949; doi:10.1038/s41746-022-00580-2)
Supplement: Supplementary file 3 — Reporting Summary Checklist [file 41746_2022_580_MOESM3_ESM.pdf]

nature portfolio

Corresponding author(s): Emily B Brant MD MS

Last updated by author(s): Feb 3, 2022

Reporting Summary

Nature Portfolio wishes to improve the reproducibility of the work that we publish. This form provides structure for consistency and transparency in reporting. For further information on Nature Portfolio policies, see our Editorial Policies and the Editorial Policy Checklist.

Statistics

For all statistical analyses, confirm that the following items are present in the figure legend, table legend, main text, or Methods section.

n/a	Confirmed	
☒	☐	The exact sample size (n) for each experimental group/condition, given as a discrete number and unit of measurement
☒	☐	A statement on whether measurements were taken from distinct samples or whether the same sample was measured repeatedly
☒	☐	The statistical test(s) used AND whether they are one- or two-sided<br>*Only common tests should be described solely by name; describe more complex techniques in the Methods section.*
☒	☐	A description of all covariates tested
☒	☐	A description of any assumptions or corrections, such as tests of normality and adjustment for multiple comparisons
☒	☐	A full description of the statistical parameters including central tendency (e.g. means) or other basic estimates (e.g. regression coefficient) AND variation (e.g. standard deviation) or associated estimates of uncertainty (e.g. confidence intervals)
☒	☐	For null hypothesis testing, the test statistic (e.g. F, t, r) with confidence intervals, effect sizes, degrees of freedom and P value noted<br>*Give P values as exact values whenever suitable.*
☒	☐	For Bayesian analysis, information on the choice of priors and Markov chain Monte Carlo settings
☒	☐	For hierarchical and complex designs, identification of the appropriate level for tests and full reporting of outcomes
☒	☐	Estimates of effect sizes (e.g. Cohen's d, Pearson's r), indicating how they were calculated

Our web collection on statistics for biologists contains articles on many of the points above.

Software and code

Policy information about availability of computer code

Data collection	No data software was used in this analysis.
Data analysis	No data software was used in this analysis.

For manuscripts utilizing custom algorithms or software that are central to the research but not yet described in published literature, software must be made available to editors and reviewers. We strongly encourage code deposition in a community repository (e.g. GitHub). See the Nature Portfolio guidelines for submitting code & software for further information.

Data

Policy information about availability of data

All manuscripts must include a data availability statement. This statement should provide the following information, where applicable:

- Accession codes, unique identifiers, or web links for publicly available datasets
- A description of any restrictions on data availability
- For clinical datasets or third party data, please ensure that the statement adheres to our policy

Data supporting the findings of this article are publicly available and cataloged in eTable 3 in the Supplement.

Field-specific reporting

Please select the one below that is the best fit for your research. If you are not sure, read the appropriate sections before making your selection.

☐ Life sciences ☒ Behavioural & social sciences ☐ Ecological, evolutionary & environmental sciences

For a reference copy of the document with all sections, see nature.com/documents/nr-reporting-summary-flat.pdf

Behavioural & social sciences study design

All studies must disclose on these points even when the disclosure is negative.

Study description	We performed a systematic review to identify clinical variables required to define sepsis and its subtypes to produce a concept mapping of elements onto Fast Healthcare Interoperability Resources (FHIR).
Research sample	Relevant papers described consensus sepsis definitions, provided criteria for sepsis, severe sepsis, septic shock or detailed sepsis subtypes.
Sampling strategy	Titles and abstracts identified through database searches were independently screened by two reviewers (E.B. and J.K.). Full text versions were then obtained and independently reviewed by co-authors. Reference lists from selected manuscripts were scrutinized by two authors (E.B. and J.K.) to identify additional studies meeting selection criteria in a snowball strategy. Sample size was not predetermined; manuscripts were included in the analysis until saturation (i.e., no additional unique variables were collected) was achieved.
Data collection	Two authors (E.B. and J.K.) collected study characteristics (e.g., title, authors, year of publication, journal, study design), population characteristics (e.g., demographics, admission diagnosis, level of care), clinical variables (e.g., vital signs, laboratory measurements), organ support (e.g., provision of mechanical ventilation or dialysis), treatments (e.g., corticosteroids), and outcomes (e.g., 28-day mortality, intensive care unit admission, hospital and/or ICU length of stay) using a standardized data extraction form developed in Microsoft Word (eTable 1). The final list of clinical variables was independently reviewed by 4 members of the study team (E.B., J.K., V.L., and C.S.; eTable 2). The final list was also reviewed with the stakeholder group (eCollaborators) to adjudicate the final list of variables for completeness and applicability.
Timing	Searches were conducted from June 2019 to January 2020.
Data exclusions	Exclusion criteria were pre-established. These included manuscripts that were i.) not English language, ii.) not available in full text, iii.) published prior to 1970, iv.) reported on pediatric populations or animals and v.) considered "grey literature" including letters, editorials, commentaries, lectures and addresses
Non-participation	Because we performed a systematic review, no participants were involved in our analysis.
Randomization	Because we performed a systematic review, no participants were involved in our analysis.

Reporting for specific materials, systems and methods

We require information from authors about some types of materials, experimental systems and methods used in many studies. Here, indicate whether each material, system or method listed is relevant to your study. If you are not sure if a list item applies to your research, read the appropriate section before selecting a response.

Materials & experimental systems

n/a	Involved in the study	
☒	☐	Antibodies
☒	☐	Eukaryotic cell lines
☒	☐	Palaeontology and archaeology
☒	☐	Animals and other organisms
☒	☐	Human research participants
☒	☐	Clinical data
☒	☐	Dual use research of concern

Methods

n/a	Involved in the study	
☒	☐	ChIP-seq
☒	☐	Flow cytometry
☒	☐	MRI-based neuroimaging
